# Smart nanoparticles for cancer therapy

**DOI:** 10.1038/s41392-023-01642-x

**Published:** 2023-11-03

**Authors:** Leming Sun, Hongmei Liu, Yanqi Ye, Yang Lei, Rehmat Islam, Sumin Tan, Rongsheng Tong, Yang-Bao Miao, Lulu Cai

**Affiliations:** 1 Personalized Drug Therapy Key Laboratory of Sichuan Province, Department of Pharmacy, Sichuan Provincial People’s Hospital, School of Medicine, University of Electronic Science and Technology of China, Chengdu, 610072 China; 2 School of Life Sciences, Engineering Research Center of Chinese Ministry of Education for Biological Diagnosis, Treatment and Protection Technology and Equipment in Special Environment, Northwestern Polytechnical University, Xi’an, 710072 China; 3 Sorrento Therapeutics Inc., 4955 Directors Place, San Diego, CA 92121 USA; 4 Department of Haematology, Sichuan Provincial People’s Hospital, School of Medicine, University of Electronic Science and Technology of China, Chengdu, 610072 China

**Keywords:** Nanobiotechnology, Cancer therapy

## Abstract

Smart nanoparticles, which can respond to biological cues or be guided by them, are emerging as a promising drug delivery platform for precise cancer treatment. The field of oncology, nanotechnology, and biomedicine has witnessed rapid progress, leading to innovative developments in smart nanoparticles for safer and more effective cancer therapy. In this review, we will highlight recent advancements in smart nanoparticles, including polymeric nanoparticles, dendrimers, micelles, liposomes, protein nanoparticles, cell membrane nanoparticles, mesoporous silica nanoparticles, gold nanoparticles, iron oxide nanoparticles, quantum dots, carbon nanotubes, black phosphorus, MOF nanoparticles, and others. We will focus on their classification, structures, synthesis, and intelligent features. These smart nanoparticles possess the ability to respond to various external and internal stimuli, such as enzymes, pH, temperature, optics, and magnetism, making them intelligent systems. Additionally, this review will explore the latest studies on tumor targeting by functionalizing the surfaces of smart nanoparticles with tumor-specific ligands like antibodies, peptides, transferrin, and folic acid. We will also summarize different types of drug delivery options, including small molecules, peptides, proteins, nucleic acids, and even living cells, for their potential use in cancer therapy. While the potential of smart nanoparticles is promising, we will also acknowledge the challenges and clinical prospects associated with their use. Finally, we will propose a blueprint that involves the use of artificial intelligence-powered nanoparticles in cancer treatment applications. By harnessing the potential of smart nanoparticles, this review aims to usher in a new era of precise and personalized cancer therapy, providing patients with individualized treatment options.

## Introduction

Cancer is a significant public health issue with a rapidly growing incidence and mortality worldwide,^1^ leading to about 10 million deaths annually.^2^ Chemotherapy is presently one of the most common anti-cancer treatments due to its high efficiency currently.^3,4^ However, its lack of selectivity for tumor cells and challenges in efficient drug delivery to the tumor site have led to practical limitations. Additionally, multi-drug resistance poses another obstacle to successful chemotherapy. The complexity of the tumor microenvironment and individual variations further contribute to the difficulty of developing effective treatment options.^5,6^ To overcome these issues, the development of new drug delivery strategies has been prompted.

Smart nanoparticles (NPs) have emerged as a promising alternative to conventional nanoparticles for cancer therapy. Unlike conventional nanoparticles, they can be triggered by specific stimuli and target-specific sites with precise drug delivery.^7,8^ After modification or stimulation by corresponding factors, these smart nanoparticles efficiently aggregate at the target location and release their payloads, establishing a smart treatment mode.^8–12^ Furthermore, their capability to co-delivering therapeutics and diagnostic reagents, which have greatly promoted the development of theranostics and smart nanoparticles for cancer therapy.^13^

**Fig. 1:**
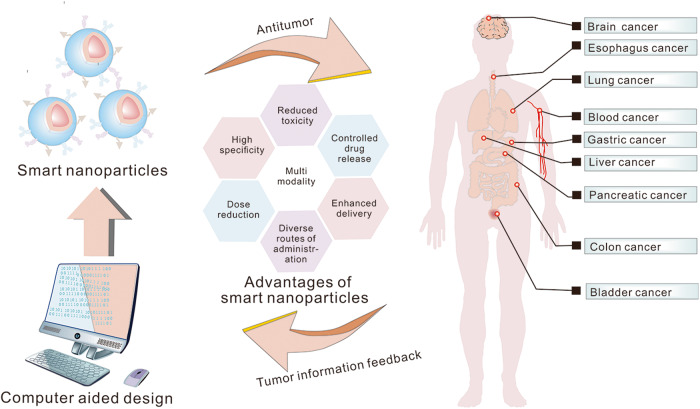
Schematic illustration of smart nanoparticles for cancer treatment

Understanding smart nanoparticles requires a variety of perspectives and perspectives that overlap. One may compare a smart nanoparticle to a toolbox in this analogy. It has the capability of modifying the size, shape, surface qualities, targeting, and composition of smart nanoparticles in response to both endogenous and exogenous stimuli produced by the cell (Fig. 1). According to the type and application of nanoparticles, we can interpret from the different types of nanocarriers, stimulating factors, target modifications and payload drugs: 1) Different nanocarriers have different structures and properties, and suitable nanocarriers can be selected according to the nature of the drug delivered and the needs of the treatment. For example, micelles are suitable for the delivery of water-insoluble and amphiphilic drugs, and liposomes can increase the cellular uptake of a variety of drugs.^14,15^ 2) Smart nanoparticles based on specific materials and components of nanocarriers can respond to external and internal stimuli, such as enzyme, pH, temperature, as well as optical and magnetic regulation, etc. 3) Another embodiment of smart nanoparticles is their tumor targeting characteristics by functionalizing their surface with tumor-specific ligands (such as antibodies, peptides, aptamer, and transferrin, etc.). 4) Unlike traditional nanoparticles that are used to deliver chemotherapeutic agents, the new generation of intelligentized nanoparticles can also deliver different types of drugs, including small molecules, peptides and proteins, nucleic acids and living cells. Additionally, the proposal of computer-aided design smart nanoparticles, integrating the cutting-edge application of artificial intelligence, further elevates the potential and sophistication of these ingenious nanoscale technologies. This review comprehensively explores the multifaceted nature of smart nanoparticles, akin to a versatile toolbox of dynamic capabilities, with boundless potential to revolutionize drug delivery and cancer treatment, ushering in a new era of precision medicine.

## Type of smart nanocarriers

**Fig. 2:**
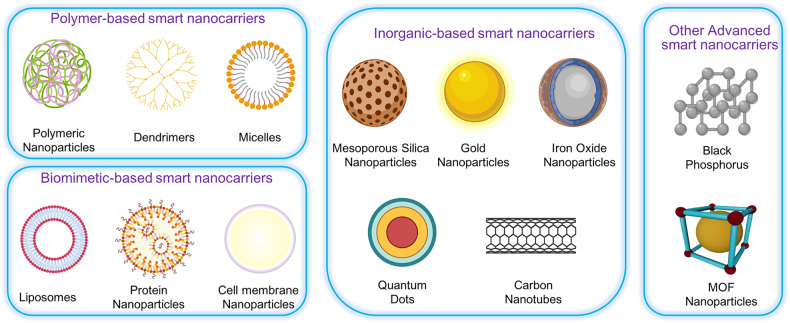
Nanocarriers for smart nanoparticles

The nano-scale drug carriers are fundamental to smart nanoparticles. To be qualified as an ideal smart nanoparticle, it should meet some basic criteria, such as stimulus response material or structure, stable nanometer size, adjustable surface charge, high encapsulation capacity, biocompatibility, degradability and low toxicity, etc. The following emphasizes the structures, classification, synthesis and smartness of the thirteen most reported nanocarriers (Fig. 2): polymer-based smart nanocarriers including polymeric nanoparticles, dendrimers, micelles; biomimetic-based smart nanocarriers including liposomes, protein nanoparticles, cell membrane nanoparticles; inorganic-based smart nanocarriers including mesoporous silica nanoparticles, gold nanoparticles, iron oxide nanoparticles, quantum dots, carbon nanotubes; other advanced smart nanocarriers including black phosphorus and metal-organic frameworks.

### Polymer-based smart nanocarriers

#### Polymeric nanoparticles

The polymeric nanoparticles with unique properties play an important role in biomedical applications, bringing biologists, chemists, engineers, and doctors together in unique collaborative ways. The development of polymeric nanoparticles represents a medical revolution that has led to major biotechnological advances in drug delivery, tissue engineering, biomaterials, and medical device development.^16,17^ The revolution demonstrated the discovery of more effective treatments in the form of proteins, nucleic acids, and other biologically active molecules.^18^ Polymeric nanoparticles have several advantages over non-mixed drugs, in terms of cycle time, stability, structural decomposition, encapsulation rate, premature release, and nonspecific release kinetics. Other advances involve the capability of combining materials with different chemical compositions such as organic-organic and organic-inorganic materials to achieve synergistic properties. Currently, the most used techniques for polymeric nanoparticles preparation are emulsion polymerization, solvent evaporation, salting-out, dialysis and supercritical fluid technologies. Research and development in the aspects of synthesis methods, manufacturing process, and mathematical models require the investigation of the mechanisms of the controlled drug release process. It enables the creation of tunable polymeric nanoparticle delivery systems that can sustain drug delivery and contribute to improved drug treatment indices. The ability to control therapeutic drug release and pluripotency of polymeric drug delivery platforms provide many important advantages. So far, we have already witnessed the first small molecule-based polymeric nanoparticles for drug targeting and controlled release from the bench to the bedside, which laid the foundation for a variety of ongoing phase II clinical trials to proceed from the initial proof of concept in vitro to the in vivo study, and to human testing.^19^

A drug delivery system can be triggered by controlling local induction of endogenous physical parameters such as electrical, thermal, ultrasound, or magnetic energy. Therefore, there is increasing interest in adding biological response elements to the entire polymer design to achieve better biologically controlled therapeutic effects. For example, poly (D, L-lactic-co-glycolic acid) can be used to intercept most types of therapeutic drugs of various molecular weights and can be made into particles of various shapes and sizes. The drug release capacity of poly (D, L-lactic-co-glycolic acid) can be adjusted by changing molecular weight, lactide to ethyl ester ratio, and drug concentration. Additional methyl groups in the polylactic acid side chain make the polymer more hydrophobic than polyglutamic acid. The increase in polylactic acid content leads to less water absorption, thus reduces the rate of degradation.^20^

Polymeric nanoparticles can be synthesized with a combination of inorganic components, such as carbon nanotubes, polymers, silica, metal oxide nanoparticles, and graphene. On the other side, organic compounds (lipids, proteins, and phospholipids) can be mixed with natural or synthetic polymers to produce new polymeric nanoparticles to achieve advantages over non-mixed drugs.^21^ The combination of multiple materials can change their respective properties and produce a mixture of materials with new unique characteristics. They can change biological distribution, solubility and improve system stability. The materials’ ability to link with one another can prolong blood circulation while maintaining biological effects. For instance, synthetic tunability enables the creation of smart nanoparticles that can simultaneously serve several therapeutic or imaging goals by co-encapsulating various therapeutic compounds with various release curves.^22,23^ Furthermore, the smart polymeric nanoparticles exhibit a unique combination of properties derived from biological and synthetic materials. For example, proteins have a short half-life, low solubility and immunogenicity, and poor stability. Protein-polymer conjugates polyethylene glycol (PEG) have improved their half-life, increased the physical stability, and rendered them immune-free.^24^

#### Dendrimers

Dendrimers are radially symmetrical, spherical, and nanoscale compact structures with branches or arms like trees.^25^ They consist of an inner layer and an outer layer. Functional groups that are used for drug conjugation and targeting make up the outer layer. The drug encapsulation efficiency, drug toxicity, and controlled release mechanisms are all improved in the inner layer.^26^ Dendrimers can be designed and modified in a variety of ways to produce hundreds of different molecules with specific properties and functions. The preparation method of dendrimers is called “iterative methods of synthesis” that have developed over time. There are two main approaches: “divergent” and “convergent”, which were originally used to make dendrite structures. In the first method, dendrimers grow from the inside to the outside and build up layer after layer. In the other approach, dendrimers grow from the outside until the core of dendrites is joined together.^27^ The controllable properties of dendrimers in the synthesis process make them have broad application prospects in many pharmaceutical preparations. Because of their special properties, such as nanoscale uniform size, high branching, water-solubility, internal cavities, polyvalency, and biocompatibility, dendrimers are ideal active excipients and can improve the solubility of insoluble drugs, reduce drug toxicity and increase drug potency.^28^

Some anti-cancer active pharmaceutical ingredients (API) are water-insoluble and generally have moderate lipophilic properties, such as paclitaxel (PTX), doxorubicin (Dox), 5-fluorouracil, camptothecin, and methotrexate. Poor drug solubility is also considered to be one of the major issues faced by drug delivery systems. Nearly 40% of newly developed APIs are rejected by the pharmaceutical industries without further development, mainly due to the low water solubility.^29^ When dendritic molecules are combined with the drug, it can greatly enhance the drug’s water-solubility, so that the drug can play a better role in the body without affecting its efficacy. For example, Sandra et al. synthesized a carbosilane dendrimer that increases compatibility with lipophilic cargo as well as enhances nanostructures due to the highly stable, inert and lipophilic nature of the carbosilane scaffolds.^30^ Moreover, the increased dendrites can give the drug additional effects. In the presence of the blood-brain barrier, drug delivery into the central nervous system is very hard. However, it can be achieved by linking the drug to a poly-amidoamine dendrimer to overcome the blood-brain barrier.^31,32^ Polylysine dendrimers have the potential to be biodegradable carriers and deliver cytotoxic drugs to solid tumors.^33^ Drug resistance, toxicity, and the mechanics of drug release in capsules can all be overcome using dendrimers, which make them ideal systems as smart nanoparticles for cancer therapy.^34^

#### Micelles

Polymer micelles typically range in size from tens to hundreds of nanometers.^35^ It is made up of two separate parts, the core region, which is colloidally stable, and the outer region, which is made up of solvated hydrophilic polymer chains (also known as the corona or shell region). While in a reverse micelle this arrangement can be changed to a hydrophilic core and a hydrophobic corona, micelles’ distinctive corona nucleus structure and capabilities allow them to improve the water solubility of hydrophobic compounds. Thus, polymeric micelles can contain both hydrophilic and hydrophobic medicines. There are many applications in biomedicine, such as tissue-engineered scaffolds and drug nanocarriers.^36^ Polymer micelles can simply physically encapsulate (solubilize) hydrophobic drugs in their hydrophobic core effectively, resulting in the following advantages: (i) elimination of drug side effects; (ii) Increase the water solubility of hydrophobic and insoluble drugs; (iii) Control of drug release rate; (iv) Protect drug molecules from degradation by specific media (pH, temperature). In addition, modern synthetic chemistry has made it possible to chemically link pharmaceuticals at the core of micelles and the design of smart polymer micelles with molecular specific targeting and stimulation of reactive drug release.^37^ The use of long-circulating polymer micelles to target tumors is a potential strategy in systemic cancer therapy. Small anti-tumor molecules such as paclitaxel are usually delivered by these surface-modified nanoscale vesicles. Numerous studies have shown that the enhanced permeability and retention (EPR) effects of micelles cause them to preferentially collect in leaky circulatory beds of solid tumors.^38^ Micellar solubilization can improve the stability and bioavailability of insoluble and almost insoluble drugs.^39^ The structure of some micelles can be reassembled by changes in pH, resulting in drug release. The pH difference between normal tissue/blood and the extracellular environment of the tumor is intrinsic, which has been widely used in drug sustained-release systems.^40^ For example, the transferrin receptor-targeted pH-sensitive micellar system can be utilized as smart nanoparticles to overcome multi-drug resistance and reduce side effects of the system, so as to achieve targeted delivery of tumors.^41^ The combination of folate-modified pH-sensitive system micelle loaded the anti-tumor drug doxorubicin can effectively reduce the systemic toxicity of doxorubicin, reduce the damage to heart and lung tissues, and improve its anti-tumor efficacy.^42^ In the stimulation-reactive nano-drug delivery system, pH-sensitive and thermosensitive polymer micelles can take advantage of the acidic conditions of solid tumors and photothermally induced hyperthermia to achieve controlled drug release, which is exceedingly suitable for chemo-photothermal therapy.^43^ Micellar is a kind of amphiphilic solubilizer with low toxicity and good biocompatibility, which also has great potential as smart nanoparticles in eye drug delivery. Micellar eye drops are more biodegradable and biocompatible materials, which are ideal to solve the problems of eye irritation and toxicity. Ginsenosides Rb1 micelle is a new ocular drug delivery system that has a good effect in improving the bioavailability of diclofenac and other drugs.^44^ Polyoxyl 15 hydroxystearate micelle can significantly improve the antioxidant activity of myricetin in vitro and accelerate the membrane permeability for optical delivery of myricetin.^45^

### Biomimetic smart nanocarriers

#### Liposomes

Liposomes are amphipathic nanoparticles with membrane like structure based on phospholipids, which are composed of a phosphate-based hydrophilic head and a fatty acid-based hydrophobic tail. The cell-like structure of liposomes allows it to fuse with cell membranes, and intelligently improve the cellular uptake of drugs. Medications that are lipid-soluble can be imbedded in lipophilic membranes, while medications that are water-soluble can be trapped in the bilayer core. Multi-lamellar vesicles and uni-lamellar vesicles are two different forms of liposomes based on the number of bilayers and the size of the liposomes. Large uni-lamellar vesicles and small uni-lamellar vesicles are the two other subgroups of uni-lamellar vesicles.^46^ There are numerous ways to make liposomes, including detergent dialysis techniques, solvent injection techniques, reverse phase evaporation techniques, and thin-film hydration techniques.^47^ Conventional techniques have numerous drawbacks. Some innovative technologies, such as supercritical fluid technologies, supercritical anti-solvent techniques, and supercritical reverse-phase evaporation techniques, have been developed to overcome those restrictions.^48^

Traditional liposomes have a number of issues, including as instability, inadequate drug loading, rapid drug release, and shorter blood circulation durations. Functionalization of conventional liposomes could make them smarter. PEG ylation helps liposomes escape the reticuloendothelial system and have a longer blood circulation time.^49^ Smart liposomes that have been functionally changed are also sensitive to a variety of internal and external stimuli, such as enzyme transformations, pH changes, redox reactions, microwaves, ultrasound, and light.^50,51^ A liposome that has been radioligand functionalized is referred to as a radiolabeled liposome. Radiolabeled liposomes can be used to both identify the tumor and treat it while also determining the bio-distribution of liposomes in the body.^52^ For example, Hansen et al. prepared a ^64^Cu-labeled liposome (^64^Cu-liposome) that can be used for combined PET/CT imaging in rats and dogs, and can achieve the chemotherapy effect of Caelyx loaded liposomes.^53^ Liposomes are potential as smart nanoparticles in the co-delivery of chemotherapeutic medicines, imaging agents, gene agents, or anticancer metals in addition to delivering imaging agents alongside chemotherapeutics.^54^ Lipid nanoparticle (LNP) have been found to co-delivery of Cas9 mRNA, focal adhesion kinase (FAK) siRNA, and sgRNA to improve both tumor delivery and gene editing efficacy.^55^

#### Protein nanoparticles

In addition to being present in soy, milk, cereals, and proteins can also be found in egg white, bovine serum, and human serum. Protein-based nanoparticles provide a number of benefits, including simple synthesis, a high binding capacity for different medications, non-toxicity, non-immunogenicity, biocompatibility, biodegradability, and plasma half-life.^56–58^ Protein nanoparticle surfaces have functional groups that make it simple to bind targeted ligands and other surface alterations as smart nanoparticles.^56,59,60^ Albumin is one of the most important proteins in plasma and has been used in various therapeutic applications over the past few decades. When used against neuroblastoma cell lines, dox-loaded human serum albumin nanoparticles were found to have superior in vitro anticancer activity to the pure drug.^61^ The successful targeting of human prostate cancer cell lines by PTX-loaded bovine serum albumin nanoparticles, which are created using a dissolving process and decorated with folic acid, has been observed.^62^ Through advantageous, noncovalent reversible binding, protein nanoparticles act as a natural transporter of hydrophobic compounds, facilitating their transit in bodily fluids and release at the cell surface. Additionally, protein can interact with the glycoprotein receptor and facilitate the transcytosis of molecules that are bound to albumin.^63^ The first commercially available drug licensed by the Food and Drug Administration (FDA) that has demonstrated notable efficacy in the treatment of metastatic breast cancer is called Abraxane, which has a diameter of about 130 nm.^12^

#### Cell membrane nanoparticles

Conventional nanoparticles continue to face a number of difficulties, including quick blood circulation clearance, simple immune system recognition, and low target site accumulation.^64^ Due to the numerous proteins that are present on the surface of cell membranes, cell membrane coating has become recognized as a viable means of overcoming these limitations.^64,65^ Cell membrane-coated nanoparticles (CMCNPs) are a biomimetic technique used to create therapeutic devices that have a nanoparticle core covered with a membrane generated from various cell sources, including cancer cells, stem cells, platelets, or white blood cells.^66,67^ The common method for the preparation of CMCNPs is the isolation of plasma membrane from different cell sources and incorporation of core nanoparticles into membrane vesicles. Due to the properties of their customized nanomaterials and advanced smart nanoparticles for cancer therapy, these biomimetic CMCNPs have recently attracted a lot of interest.^68^ For instance, platelet membrane-coated nanoparticles with Dox loaded into the inner nanoparticles and tumor necrosis factor (TNF)-related apoptosis-inducing ligand (TRAIL) put onto the outer membrane have been successfully created. In an animal model with a subcutaneous tumor and a metastatic site, the results demonstrated that platelet membrane-coated nanoparticles exhibited the strongest anticancer activity.^69^

### Inorganic smart nanocarriers

#### Mesoporous silica nanoparticles

Mesoporous materials contain pores with diameters between 2 and 50 nm, as defined by the International Union of Pure and Applied Chemistry (IUPAC).^70^ In order to refer to zeolite-silica gel compositions with clearly defined and consistent porosity, the term mesoporous silica nanoparticle (MSN) was first used forty years ago. Because of their homogeneous and adjustable pore size (2–6 nm), tunable particle size (50–300 nm), large surface area, high pore volume, and biocompatibility, MSNs are extensively investigated. A smart nanocarrier must have configurable pore size and tunable particle size, which enable the loading of pharmaceuticals with various molecular forms. For grafting various functional groups on MSNs, the high surface areas of the interior surface (pores) and external surface are ideal. Nanocarriers are a great option since they adhere to cancer cells through the EPR effect and are biocompatible.^71^ There are mainly two types of MSNs, mesoporous silica nanoparticles, and hollow or rattle-type mesoporous silica nanoparticles.^72^ Both the soft template method and the hard template method can be used to create those MSNs.

Due to hemolysis of human red blood cells, nonspecific binding to human serum protein, and phagocytosis of macrophages produced from the human monocytic leukemia cell (THP-1) line, conventional MSNs have short blood circulation half-lives. PEGylation can produce stealth behavior, helping to counteract such effects.^73^ Grafting co-polymers onto the surfaces of smart MSNs enables control over their pore apertures. Gatekeepers are grafted co-polymers. The poly(*N*-isopropylacrylamide) grafted hollow MSN allows the nanochannels access the internal hollow reservoir to be switched between “open” and “closed” states by regulating the temperature, allowing on-demand loading and releasing of small molecules.^74^ Mesoporous silica nanoparticles can have their surface altered for active targeting by adding peptides, folate, mannose, and transferrin.^75–77^ Smart MSNs have the ability to release the loaded medications in response to a variety of stimuli, including redox reaction, pH change, magnetic field, temperature change, enzyme transformation, and light.^78^

#### Gold nanoparticles

Numerous nanoparticles made from various bulk elements, including gold, silver, copper, iron, platinum, cobalt, etc., have been generated as a result of the ongoing development of nanotechnology and medical science.^79^ These elements are synthesized by biological or physicochemical methods. Due to their simple production, high specific surface area, surface plasmon resonance, stable characteristics, surface chemistry, and multi-functionalization, gold nanoparticles are considered as having tremendous potential in the diagnosis of many malignancies and medication delivery. Furthermore, the non-toxic, non-immunologic, highly permeable, and retention effects of gold nanoparticles make them more likely to infiltrate into the tumor site and to produce better therapeutic effects.^80^ Physical techniques (laser ablation, microwave and ultraviolet (UV) irradiation), chemical, and biological techniques are the main ways to create gold nanoparticles.^81^ Under specific circumstances (pH, temperature), chemical procedures typically make use of chemicals and solvents that are hazardous to the environment and people’s health. Biosynthesis (plant and microbial mediated) of gold nanoparticles has a very broad prospect, and many medicinal plants and microorganisms have the potential to produce nanoparticles in bulk.^82^ A variety of gold nanoparticles, including gold nanorods, nanostars, nanocubes, nanocages, and nanospheres, among others, have exceptional optical and physical qualities that make them particularly useful in the detection and treatment of cancer.^83^ They are appealing for targeted drug delivery, photothermal therapy (PTT), photodynamic therapy (PDT), photoimaging, biosensors, and photothermal therapy.^84^ Due to their biological inertness and capacity to give superior spatial and temporal resolution for imaging, gold nanoparticles (nanorods, cages, and shells) are regarded as the best optical imaging nanoparticles for cancer treatment. Injects millions of functional gold nanoparticles into tumors at specific time points. Upon injection, gold nanoparticles bind specifically to cancer cells and scatter shine, allowing the doctors to easily identify tumors and healthy cells.^85^ As a result of gold nanoparticles’ near-infrared absorption, interest in PTT has grown recently.^86^ Chang et al. proposed a multifunctional nano platform based on Ti3C2-MXene Au nanocomposite, which realized the triune of PTT/Enzyme kinetics therapy/antitumor immune therapy, and accompanied by photoacoustic (PA) and thermal dual-mode imaging in vivo.^87^

Gold nanoparticles have a large surface area and can be utilized to load or bind to any genetic or biological part, thereby widely used for sensing/imaging/therapy of various targets such as proteins, cells and nucleic acids.^88^ Additionally, gold nanoparticles are amenable to modification due to their negative charge on the surface, ease of synthesis, controllability of size and shape, and ability to regulate surface chemistry. As a result, they can be easily functionalized by adding a variety of biomolecules, such as drugs, targeted ligands, amino acids, and genes, making them useful smart nanoparticles for biomedical applications. For example, gold nanoparticles co-modified with glutamine and lysine can generate tumor-specific photothermal therapy by in situ generation of photothermal agents through an intra-tumor enzyme-catalyzed reaction.^89^ Gold nanoparticles labeled with tumor-homing peptide containing isoDGR can be targeted delivery of therapeutic agents to tumors and improve its therapeutic index.^90^ It’s crucial to note that the surface and core characteristics of gold nanoparticles can be tailored for specific and varied applications, such as molecular recognition, chemical sensing, drug administration, and imaging.^91^

#### Iron oxide nanoparticles

Iron oxide nanoparticles with core sizes between 10 and 100 nm include the small manmade minerals magnetite and maghemite. Iron oxide nanoparticles that have been mixed with transition metals including copper, cobalt, and nickel are also included in this group. Super para-magnetism is a peculiar phenomenon that occurs when magnetic nanoparticles are shrunk to 10–20 nm. Iron oxide nanoparticles are magnetized to their saturation when a magnetic field is applied, but there is no residual magnetism after the magnetic field is removed.^92^ Therefore, iron oxide nanoparticles have been applied for enhancing the contrast in magnetic resonance imaging. There are several ways to make iron oxide nanoparticles, including thermal decomposition, co-precipitation, hydrothermal, sono-chemical, micro-emulsion, and microwave-assisted synthesis techniques.^93^ The one that dominates among them is chemical synthesis.

Targeted medication delivery using stimuli-responsive polymer-coated iron oxide nanoparticles is a hot topic of research. Phase, solubility, and hydrophobicity conformation changes are examples of physical and chemical transitions experienced by responsive polymers. Iron oxide nanoparticles coated with polymers respond differently to temperature variations and pH gradients, according to a recent study.^94,95^ An external magnetic field has the ability to regulate these smart nanoparticles. Since nucleic acids have a negative charge due to the presence of the phosphate group, cationic lipids and polymers can be added to iron oxide nanoparticles to carry genetic material.^96^ Therefore, iron oxide nanoparticles are thus part of the group of smart nanoparticles with theranostic capabilities.

#### Quantum dots

With their distinctive photophysical properties, quantum dots (QDs) are frequently constructed from hundreds to thousands of atoms of group II and group VI molecules.^97^ The tumor might be seen while the medicine is being released at the desired location using this nanoparticle.^98^ Three components make up the majority of commercially available QDs: a core, a shell, and a capping substance. Materials used in semiconductors make up the core. Shells are constructed around the semiconductor core using ZnS. The two layer QDs are enclosed in a cap made of various materials.^99^ For a number of reasons, QD-based smart nanoparticles have generated a lot of attention. First, the core size of a QD is quite small, measuring between 2 and 10 nm in diameter. It can be used as a tracer in other drug delivery systems because of this characteristic. Second, flexible surface chemistry enables a range of methods for QD surface modification. Third, QDs can monitor drug release and drug-carrying in real time because to their photophysical capabilities.^100^ Either a top-down strategy or a bottom-up strategy can be used to synthesize QDs. Top-down processing techniques include molecular beam epitaxy, ion implantation, e-beam lithography, and X-ray lithography.^101^ However, colloidal QDs are made by a bottom-up method called self-assembly in solution after chemical reduction.^102^

The functionalization of conventional QDs, like that of other smart nanoparticles, is of equal importance.^103^ QDs are also taken up by the reticuloendothelial system non-specifically, as has been observed for other nanoparticles. Without a targeting ligand, properly PEGylated QDs can accumulate in tumor locations via the EPR effect. Various ligands, including peptides, folate, and big proteins, can be grafted on the QD surface to actively target a tumor location.^104^ As a result of their innate fluorescence, QDs are particularly well-known for cancer imaging. With CISe as the core, ZnS as the shell, manganese doping, and folic acid functionalization, a multifunctional QD has recently been produced. It possesses high near-infrared (NIR)-II fluorescence efficiency of up to 31.2% and high contrast on magnetic resonance imaging (MRI).^105^

#### Carbon nanotubes

In the form of hollow spheres, ellipsoids, tubes, and many other shapes, carbon nanotubes (CNTs) are a type of fullerene, a class of carbon allotropes.^106^ A CNT is a graphene sheet that has been wound up into a seamless cylindrical tube. Single-walled CNTs (SWCNTs) and multi-walled CNTs (MWCNTs) are the two varieties of CNTs.^107^ As a result of the CNT’s significant optical absorption in the near-infrared (NIR) region, this particle is an excellent candidate for photo-thermal ablation. Furthermore, nanoparticles with diameters between 50 and 100 nm are easily absorbed. MWCNTs are able to cross the barriers of different cellular compartments. A particular cellular compartment can be localized using PEGylated SWCNTs. Carbon black and graphite can be heated in a controlled flame environment to create CNTs. The size, mechanical strength, quality, and purity of the synthesized CNTs, however, cannot be controlled by this procedure. Electric arc discharges, chemical vapor deposition techniques, and laser ablation techniques have been described to address the constraints of the controlled flame environment.^108^ SWCNTs are more effective at delivering drugs than MWCNTs because their walls are more clearly defined and MWCNTs tend to have more structural flaws.^109^ Due to their excellent thermodynamic and optical properties, they are now regarded as one of the most promising materials for cancer sensing, bioimaging and therapeutics.

To provide CNTs smart properties, they can be chemically or physically functionalized.^110^ To boost solubility, circumvent the reticuloendothelial system, and reduce toxicity, PEGylation is a crucial step.^111^ For instance, the cyclosporin A (CsA) was conjugated to an amine-terminated phospholipid–PEG chain attached on SWCNTs via a cleavable ester bond and demonstrated the possible potential of PEGylated SWCNT-based systems for CsA delivery.^112^ Recent studies exhibited that functionalized CNTs can overcome the blood-brain barrier.^113^ CNTs have shown promise in carrying plasmid DNA, small interfering ribonucleic acid, antisense oligonucleotides, and aptamers.^114^ It can be utilized for thermal ablation of cancer areas in addition to gene delivery.^115^ As diagnostic instruments for the early identification of cancer, functionalized CNTs can also be used.^116^

### Other advanced smart nanoparticles

Except for the above smart nanoparticles that could be a benefit for cancer therapy, there are also some recent developed advanced smart nanoparticles such as black phosphorus (BP), metal-organic framework (MOF), topologically heterogeneous nanoparticles and so on that have attracted more and more attention due to their unique properties and great potential for cancer therapy.

#### Black phosphorus

Due to its distinct physical, chemical, and biological qualities, BP was originally created in 1914 and has since garnered a lot of interest.^117^ The most stable allotrope of phosphorus is BP. Individual phosphorus atoms are in the sp^3^ hybridization state in BP, which results in wrinkled layers that are stacked vertically and attracted to one another by weak van der Waals interactions. Traditionally, BP can be prepared by mineralization routes, high-pressure routes, and mechanical milling techniques.^118^ High-energy mechanical milling (HEMM) is the most commonly utilized method for the fabrication of BP nanoparticles, which have excellent biocompatibility and biodegradability for biomedicine.^119^ It is widely known that BP has outstanding photothermal properties when exposed to NIR radiation, which opens up a wide range of applications for it as smart nanoparticles for cancer photoacoustic (PA) imaging and PTT.^120^ For instance, the solventless HEMM approach was used to successfully prepare water-soluble and biocompatible PEGylated BP nanoparticles with a high yield. The resulting PEGylated BP nanoparticles have a homogeneous size, high biocompatibility, photostability, and the capacity to generate heat from NIR light, making them appropriate as a novel nanotheranostic agent for photothermal therapy and PA imaging of cancer.^121^

#### MOF nanoparticles

MOFs are made of organic molecules that act as linkers and one or more types of metal ions.^122^ Due to the versatility of its chemical composition, there are many types of MOFs such as zeolite-like structures (ZIF) using imidazoles as ligands, polymorphism in MOFs (MIL), MOFs with alkaline earth metals (AEPF), and MOFs with rare earth as the metal center (RPF) using benzenecarboxylated acids (HKUST).^122^ The solvothermal method, coprecipitation methods, mechanochemical methods, microwaves, and sonochemical methods are the common methods for the preparation of MOFs.^122^ As hybrid crystalline porous biomaterials, many attempts have been made to utilize MOFs as smart nanoparticles in drug delivery systems due to their adjustable pore shape and size, ultrahigh surface area, and versatile functionalities.^123^ However, the limitations of physiological instability and the cytotoxicity of MOFs from toxic metal ions have limited their drug delivery applications.^124^ As a result, combining MOFs with functional materials provides a novel approach to creating multifunctional hybrids for cancer therapies like PDT, PTT, immunotherapy, chemotherapy, and combination therapy, among others.^125^ An endogenous copper metal-organic framework nanoenzyme has been demonstrated to mediate the synergistic interaction between H_2_S-activated NIR PTT and chemodynamic therapy in the successful treatment of colon cancer, according to a recent study. By avoiding the introduction of cargo, this endogenous biomarker-triggered “turn-on” technique to produce therapeutic molecules in situ could greatly simplify the construction of nanomedicine and hold significant promise for the targeted treatment of colon cancer.^126^

#### Topologically heterogeneous nanoparticles

Topologically heterogeneous nanoparticles including many different types, such as core-shell nanoparticles, Janus nanoparticles, hybrid nanoparticles and so on.^127^ The core-shell nanoparticles have a core region and a shell region, where each region can have different properties. For example, the core can encapsulate the therapeutic agent, while the shell can provide stability, control release, or offer targeting ligands.^128^ Janus nanoparticles are composed of two distinct regions or materials, often with different physicochemical properties. These nanoparticles can be designed to have different drug-loading capacities, surface functionalities, or release profiles on each side.^129^ Hybrid nanoparticles combine different materials or structures to create topological heterogeneity. For example, a nanocarrier might incorporate both polymeric and lipid components, each with unique characteristics to optimize drug loading, stability, and targeting.^130^ The topological heterogeneity nanoparticles offer several advantages for drug delivery in cancer therapy. It allows for precise control over drug loading, release kinetics, and targeting efficiency.^131^ These advanced nanoparticles with topologically heterogeneous structures have promise for overcoming biological barriers, improving therapeutic efficacy, and minimizing side effects in cancer therapy.^132^

## Stimuli

**Fig. 3:**
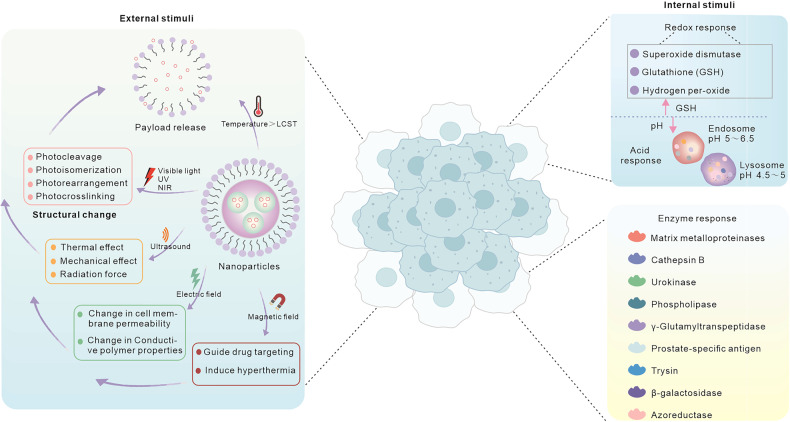
The endogenous and exogenous stimuli of smart nanoparticles for cancer therapy

The smart nanoparticle functions much like a toolkit. Smart nanoparticles can modify their form, structure, solubility, surface charge, self-association, or dissociation behaviors in response to internal and external stimuli (Fig. 3), which can increase endosomal escape, promote cellular uptake, or cause payload release.^133^ There are two ways to make nanoparticles respond to internal or external stimuli: first, to use nanomaterials with responsive effects, such as gold nanoparticles sensitive to light and heat; second, to modify the nanoparticles by polymerizing or linking functional groups with responsive effects.^134,135^ Next, we will explain the principles of a variety of endogenous and exogenous stimuli used in smart nanoparticles.

### Endogenous stimuli

#### pH

The pH-responsive nanoparticles against the tumor extracellular and intracellular stimuli are the most widely investigated to date. Almost all solid tumors are prone to speeding up the rate of glycolysis by generating a bulking amount of lactic acid to provide adequate energy for tumor cells. This truncated pathway is also known as the Warburg effect of converting glucose directly into lactic acid.^136^ Therefore, the acidic microenvironment of the tumor is the origin of the design of pH-responsive smart nanoparticles.

The extracellular pH of tumor mass is slightly acidic between 6.5 to 7 and the intracellular pH is slightly higher than normal tissues and other biological fluids. However, some intracellular compartments are more acidic, like endosomes (pH ~5–6.5) and lysosomes (pH ~4.5–5).^137^ Therefore, the significant pH gradient between the tumor and other physiological tissues is a possible approach to synthesize and modify pH-responsive smart nanoparticles. Generally, two basic perceptions define the mechanism of the pH-responsive nanoparticles, such as the cleavage of chemical bonds and nanocarrier protonation.^138^ Chemical bonds of pH-responsive linkers disassemble at acidic pH and release the drug in the tumor microenvironment. So far, various pH-sensitive linkers or bonds have been reported, such as hydrazones, amides, orthoesters, and vinylesters.^139^ The pH-sensitive linkers allow the conjugation of drugs and polymers through pH-dependent conformational changes to intelligently deliver the therapeutic agent in the desired location and avoid systematic toxicity.^140^ Moreover, smart pH-sensitive nanoparticles can also change their surface charge at different pH levels and the process is known as a charge reversal strategy, which can increase the cellular absorption of drugs in the low-acidic tumor environment.

#### Enzyme

Enzymes are widely present in the tissues and organs to maintain the normal operation of human body.^141^ The tumor microenvironment exhibits aberrant expression of enzymes such as matrix metalloproteinases, cathepsins, phospholipases, and oxidoreductases because tumor cells grow more quickly than other normal organs and require more enzymes for functional support.^142^ The up or down-regulation of enzymes has attracted the attention of researchers. The development of enzyme-responsive nanoparticles for targeted therapy of tumor microenvironment is another effective strategy for nanoparticle intelligence which could release the payload into the desired target.^143^ The following will exhibit several typical enzymes used in enzyme-response smart nano-therapeutic strategies.

Cancer cell invasion and metastasis have long been facilitated by matrix metalloproteinases (MMPs), whose expression and activation have risen in nearly all human malignancies.^144^ Therefore, MMPs are feasible targets for enzyme-responsive smart nanoparticle design. MMP is one of the most studied proteases in the intelligent delivery system of anti-tumor drugs. However, not all matrix metalloproteinases have tumor-promoting effects. For example, MMP-3 and MMP-8 have been found to inhibit tumor angiogenesis in skin cancer. If MMPs are used as an intelligent strategy for the treatment of cancer therapy, it is necessary to clearly understand the expression and role of individual MMPs in a specific cancer context.^145^

Cathepsin B is a cysteine protease that is widely expressed in lysosomes and is involved in the conversion of proteins there. However, studies have indicated that cancer patients have higher levels of its protein and activity.^146^ The role of cathepsin B in cells is to drive caspase-dependent apoptosis and promote tumor invasion, metastasis and angiogenesis.^147^ According to earlier research, cathepsins are most active in an acidic environment and are released in an active state when the pH of the environment around the cell is below a certain level.^148,149^ This provides a direction for the design of intelligent nanoparticles with dual response of pH and enzyme. In addition, cathepsins E, L, S, and K can also be used as targets for the enzyme response of smart nanoparticles.^150^

Urokinase is a serine protease, which is also known as urokinase plasminogen activator (uPA). The binding of uPA and its receptor (uPAR) will help activate the serine protease plasminogen to become plasmin. Afterwards, start a series of proteolytic cascade reactions to break down the extracellular matrix’s components and encourage tumor invasion and metastasis.^142,151^ Additionally, the uPA-uPAR connection triggers signals that promote tumor-promoting gene expression and cell survival and proliferation.^152^ Unlike normal tissues, uPA and uPAR levels are constitutively increased in majority of cancer types.^153,154^ thus, uPA and uPAR are often considered as attractive enzyme stimulation response target for smart nanoparticles.

Phospholipase can hydrolyze phospholipids into fatty acids and other lipophilic substances. It is overexpressed in infectious diseases, inflammation and peripheral areas of tumor invasion. Secretory phospholipase A2 (sPLA2) is the most studied, aberrant expression of human group IIA (hGIIA), III (hGIII) and X (hGX) sPLA2s has been associated with the pathology of colorectal, breast, gastric, oesophageal, ovarian and prostate cancers, which provide the potential for phospholipase to be designed as a stimulator in releasing drugs responsively.^155,156^ In addition, hyaluronidase, γ-Glutamyltranspeptidase, prostate-specific antigen, Trysin, β-galactosidase, Azoreductase, etc. are also used in enzyme-stimulated response smart nano-delivery systems.^157–162^

#### Redox

The redox-responsive system is another promising approach for endogenous stimuli-based smart delivery of therapeutic agents. Tumor cells have relatively high oxidative stress compared to healthy cells due to the presence of reactive species. The reactive oxygen (ROS) is the by-product of aerobic metabolism, including superoxide dismutase, glutathione (GSH), and hydrogen per-oxide.^163,164^ In particular, GSH is four times higher in tumors than in healthy tissues, and its intracellular concentration (2-10 mM) is thousands of times higher than in the extracellular compartment. This unique internal environment provides the feasibility of a redox-responsive smart nano-delivery system. The currently constructed smart delivery system mainly contains redox stimulants such as disulfide bonds, diselenide bonds, succinimide-thioether bonds and “trimethyl-locked” benzoquinone.^165^ Among them, the redox-responsive delivery system containing disulfide bonds that can be cleaved by GSH is studied extensively.

For the delivery of chemotherapeutic drugs to treat cancer, many redox-responsive nanoparticles have been investigated recently,^166^ including polymers, micelles, MSNs, siRNA, proteins, and liposomes for the controlled delivery system in cancer therapy.^167,168^

### Exogenous stimuli

#### Temperature

Since diseased/tumor tissues have the physiological characteristics of higher temperature (40–42 °C) than normal tissues (37 °C).^169^ One of the exogenous stimulation factors for cancer treatment that has been the subject of the most research is the temperature- or thermos-responsive medication delivery system. The payload of a thermo-responsive smart medicine carrier device typically remains at room temperature and is released only when exposed to high temperatures.

The lower CST (critical solution temperature) (LCST) and the upper CST (UCST) are the two primary properties of thermosensitive materials employed in thermally responsive smart nanosystems.^170^ Thermally responsive polymers are a key component of these systems, which are subjected to a distinctive volume phase transition to become hydrophobic or hydrophilic at different temperature.^171^ When below the LCST, the polymer becomes insoluble, above LCST, temperature-responsive polymeric nanoparticles with cellulose derivatives such as carboxymethyl cellulose (CMC) can cause a transition from hydrophilic to hydrophobic and triggers the release of the payload.

The temperature-responsive smart nanoparticles using polymeric, nanogel, and organic or inorganic materials for anti-cancer therapy have gained attention in recent years. Poly N-isopropyl acrylamide (PNIPAM) is the most widely used temperature-responsive polymer with the advantage of low LCST (32 °C) in an aqueous solution and becomes hydrophobic above LCST.^172^ And PNIPAAM copolymerization with other monomers, such as N, N-dimethyl acrylamide (DMAAm), will adjust the LCST,^173^ thereby adjusting the hydrophobic interaction and optimizing the temperature -sensitive carrier.

#### Light

The use of light in the treatment of diseases is a very significant discovery. Phototherapy is a method of preventing and curing diseases by using visible and invisible light from sunlight and artificial light sources. Since they can provide geographic and temporal control of the release of the encapsulated medicines on demand via light irradiation, photo-responsive smart nanoparticles have gained significant interest as controlled drug delivery systems in the field of cancer smart nanoparticle therapy.^174^ And light sources include visible light, UV, or NIR. The method of realizing the critical parameters for the control of structures and functions of nanomaterials are precisely dependent on the light intensity, wavelength, and exposure time

Photo-responsive smart nanoparticles are mainly by loading photosensitive components. According to the different light response mechanism, there are four kinds of light sensitive groups: 1) photocleavage group, such as o-nitrobenzyl and coumarin-based groups, etc. 2) photoisomerization group, such as azobenzenes and spiropyran. 3) photo-induced rearrangement group, such as 2-diazo-1,2-naphthoquinone. 4) photocrosslinking group, such as coumarin, cinnamoyl, anthracene, etc.^175,176^ The photocleavage type drug delivery system contains a photo-fragmentation group, which undergoes a chemical bond cleavage reaction under UV or NIR irradiation, which causes the structure of the drug delivery system to change and release the drug. The photoisomerization type drug delivery system contains a photoisomerization group. Azobenzene is a typical representative of photoisomerization molecules. Under the stimulation of UV light (300–400 nm), azobenzene can be transformed from the trans to the cis conformation, and under the stimulation of visible light (>400 nm), the transformation from cis to trans conformation. The trans-cis conformational transition of this photoisomerized molecule can cause changes in the internal structure of the corresponding drug-loaded nanocarriers, and achieve the effect of light-controlled drug release. The photorearrangement type drug delivery system contains a photorearrangement group, and its photochemical reaction changes the internal structure of the drug delivery system under light stimulation, causing the nanoparticles to swell and disintegrate to release the cargo. The drug delivery system containing photocrosslinked molecules can undergo photocrosslinking or photolysis reaction under suitable illumination, which destroys the stability of the drug delivery system to achieve light-triggered drug release. To conclude, light as an easily controllable external stimulus for the controlled release of drugs in smart nano-delivery systems is very promising.

#### Ultrasound

The role of ultrasound technology in disease diagnosis and treatment has been clinically recognized. Since it can accurately focus on a target location, ultrasound provides greater spatiotemporal control and the inherent qualities of safety, non-invasiveness, tunable frequency, and deep tissue penetration. As a result, it has become a common external trigger approach for cancer treatment.^177,178^ The biological effects of ultrasound include thermal, mechanical, cavitation, thixotropic and acoustic impulse effect. The thermal effect, mechanical effect and the radiation force generated by ultrasound enhance the transient tissue permeability and are the reasons for stimulating the release of carrier drugs.^179^ Moreover, the thermal and mechanical effect are the basis of ultrasonic-responsive smart nanoparticles.

Ultrasound-responsive smart nanoparticles have their ability to enhance ultrasonic -contrast so that they can be used for imaging diagnosis and promoting image-guided drug delivery. Additionally, the capacity to release medicines pulsatility on demand in response to ultrasound stimulation and to increase the permeability of physiological barriers such the stratum corneum, vascular endothelium, and the blood-brain barrier (BBB).^180^ In addition, since ultrasound has the characteristics of thermal response, it can be used for the construction of dual-response smart nanosystems of thermal and ultrasound.

#### Electric field

Electric field is a non-invasive stimulating factor, which helps to improve the efficiency of drug treatment. Electric fields have been used clinically for the treatment of cancer. For example, electric field treatment of glioblastoma has been approved by the US FDA, indicating that it is feasible to introduce electric fields to cancer treatment.^181^ Therefore, the electric field response system is a promising exogenous stimulation method for the intelligent delivery of cancer.

High-intensity exogenous electric fields have the ability to directly affect the permeability of cellular membranes, which can be employed as a stimulant for drug administration.^178^ It also includes triggering the conductive polymer or implantable electron transport device when an electric potential is applied, such as changing the physical properties of the conductive polymer to release the payload. Common conductive polymers include polypyrrole (PPy), polyaniline along its derivatives poly(3,4-ethylenedioxythiophene) (PEDOT), which are widely used because of their biocompatibility and ability to convert electrical energy into mechanical energy.^182,183^ Other polymer composites or conductive fillers, such as carbon nanotubes, graphene and metal nanoparticles, can also be added.^184^

#### Magnetic field

The detection and treatment of disorders, including cancer, have made extensive use of magnetic resonance imaging (MRI). Due to their biocompatibility, biodegradability, simplicity in synthesis as co-precipitates or microemulsions, and ease of customization and functionalization for particular purposes, magnetic systems have a very broad range of uses. With the rapid development of functional nanomaterials, many non-invasive and efficient cancer diagnosis and treatment methods have been developed. The target location can be easily reached by magnetic nanoparticles (MNPs), which are tiny and have a large specific surface area.^185^ MNPs are therefore anticipated to develop into a possible medication delivery mechanism. The use of magnetic external stimulation to intelligently control the release of payload in magnetic nanoparticles is a research hotspot in the field.

The MNPs in tumor tissues act as transducers in the magnetic stimulation response drug release system, converting hysteresis loss or relaxation loss into heat when an external alternating magnetic field is applied. As a result, the magnetic response intelligent nano system has two treatment procedures. The first technique uses magnetic fields to assist drug targeting, whereas the second uses magnetic fields to cause hyperthermia.^186–188^ In addition, the magnetic nanosystem can be used as a nano-scale imaging probe, which contributes to early diagnosis.^189^

Magnetic-responsive drug delivery systems usually assembled paramagnetic and superparamagnetic nanoparticles in polymer scaffolds to promote the accumulation of anti-cancer drugs in tumors under a permanent magnetic field. Among different inorganic nanomaterials, MNPs have been widely employed for tumor targeting.^190^ In particular, superparamagnetic iron oxide nanoparticles (SPIONs) have been widely used with magnetite (Fe_3_O_4_) or maghemite (Fe_2_O_3_) as a magnetic core and core-shell structure conjugated with polymer or silica on the outer surface.^191^ Iron oxide nanoparticles (IONPs) have attracted considerable interest due to their outstanding magnetic properties, biocompatibility, low toxicity, and biodegradability. These SPIONs have the advantage of producing heat in the local tumor environment by applying an external alternating magnetic field (AMF). In recent years, AMF’s role as a promising therapeutic modality that raises the localized temperature and consequently releases the loaded drug in a spatiotemporal manner has attracted significant scientific attention.^192^

## Target

**Fig. 4:**
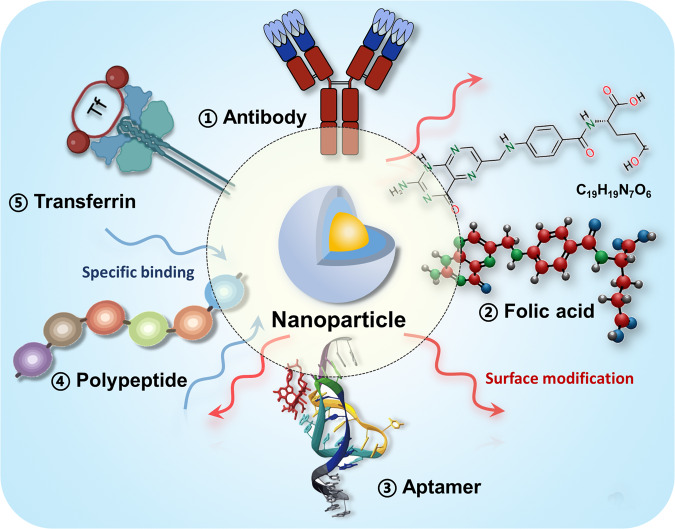
Tumor-specific target modification of smart nanoparticles categorized by aptamer, antibody, peptide, folic acid and transferrin

So far, the accumulation of nanoscale therapeutic agents at tumor sites has largely relied on the EPR effect caused by tumor vascular extravasation.^193^ A large number of passive targeting strategies based on the EPR effect have been studied to improve drug efficacy and reduce systemic toxicity. However, because tumor blood flow and vascular permeability are significantly varied from each other, the EPR effect may not be beneficial for all solid tumors.^194^ Besides, the EPR effect in humans is not as effective as in rodents. Different from passive targeting strategy, ‘missile-like’ nanoparticles for active tumor-targeting is more charming. There are a variety of specific receptors on the surface of tumor cells (such as antibodies, peptides, transferrin, folic acid, etc.). Specific ligands can be modified on the surface of nanoparticles, and the specific binding between the receptors and their corresponding ligands can realize the active targeted drug delivery (Fig. 4).

### Antibody modification

For the diagnosis and therapy of illnesses, antibodies are known target-specific reagents. In the field of smart drug targeting, active targeting based on tumor surface-specific antibodies has always attracted attention, and cancer-targeted antibodies have been clinically proven to be successful. We aim to provide a comprehensive overview of the antibodies currently used for surface modification of nanocarriers.

#### Epidermal growth factor receptor (EGFR) antibody

EGFR is a cell surface receptor expressed in many different types of cells, especially overexpressed in cancer cells.^195^ Researchers have shown that the overexpression of EGFR is closely related to the differentiation and migration of cancer cells. Cetuximab is a chimeric monoclonal antibody that selectively binds to EGFR, which can effectively block the activation of EGFR and its downstream signaling pathways, thereby inhibiting EGFR-related development and cancer progression.^196^ Cetuximab is currently the most commonly used anti-EGFR antibody for surface modification of nanocarriers. McDaid et al. used cetuximab as a targeting agent for camptothecin-loaded polymer nanoparticles to act on EGFR overexpression and cytoxan (CTX)-resistant cancer cells. The results confirmed that the CTX coupled nano drug delivery system enhanced the ability of NPs to target the cell surface by interacting with EGFR.^195^ More studies have also proven that the CTX-modified nano-drug carrier system has better targeting and exerts a stronger tumor suppressor effect.^196–198^

#### Vascular endothelial growth factor (VEGF) antibody

In the process of tumor progression, tumor vascularization provides blood supply for the rapid growth of tumors and the potential for metastasis. Among active targeting strategies, targeting tumor angiogenesis has been a research hotspot in recent years. VEGF is an angiogenic protein that is overexpressed on various tumor cells to increase the vascularization and vascular permeability of tumor tissues. Therefore, targeting VEGF has attracted great attention. The VEGF ligand is used to couple with the drug delivery system to guide the drug delivery system to accumulate on tumor tissues and adhere to tumor cells, aiming to enhance targeting and improve treatment efficiency. Liu et al. prepared VEGF (ab68334) antibody-modified paclitaxel-loaded multi-block polymer nanoparticles (VPNP), and evaluated the efficiency of target ligand coupling. Nanoparticles modified by VEGF antibody can promote the adhesion of VPNP to tumor cells and mediate internalization, showing more cell uptake and higher cytotoxicity.^199^

Vascular endothelial growth factor receptor (VEGFR) is overexpressed on the surface of a variety of tumor cells and in situ neovascularization, and it is also a potential target for tumor therapy. Liu et al. developed a VEGFR-mediated drug delivery system. They modified the lipid carrier with anti-VEGFR-2 antibody, which can effectively deliver docetaxel (DTX) to the tumor vasculature and tumor, and inhibit tumor growth.^200^

#### Human epidermal growth factor receptor (HER) antibody

Human epidermal growth factor receptor 2 (HER2) is a member of the ErbB family of receptor tyrosine kinases. It is highly expressed in about 30% of breast cancers and 20% of ovarian cancers, and is an effective target for clinical treatment of breast cancer. Chen et al. used the Fab’ fragment of humanized anti-HER2 monoclonal antibody (rhuMAbHER2) to couple with PE38KDEL-loaded polylactic acid-glycolic acid (PLGA) nanoparticles. The use of Fab’ fragments helps the antibody-modified NPs to better penetrate into solid tumors. The results also show that the modified nanoparticles have better anti-tumor activity in vivo and in vitro against breast cancer overexpressing HER2.^201^

Trastuzumab (TAB) is also a typical anti-HER2 antibody. Niza et al. used TAB to modify Dasatinib-encapsulated nanoparticles to target HER2 overexpressing breast cancer cells.^202^ Fu et al. also used TAB to modify lipopolymer hybrid nanoparticles loaded with cisplatin (CIS) and 5-fluoropyrimidine (5-FU), providing a new tumor treatment strategy with higher efficacy and fewer side effects.^203^

#### Fibroblast growth factor receptor 3 (FGFR3) antibody

FGFR3 is a transmembrane protein that is overexpressed in about 20% of advanced bladder cancers and leads to the development of aggressive tumors. At present, FDA has approved several FGFR inhibitors for the clinical treatment of cancer.^204,205^ In addition, the use of antibodies that actively bind to the FGFR3 antigen can not only achieve therapeutic effects, but also effectively target bladder cancer cells. For example, Hortelao et al. used anti-FGFR3 coupled with PEG-modified mesoporous silica nanoparticles to show higher internalization efficiency for bladder cancer multicellular spheroids and induce stronger cytotoxicity.^206^

#### P-glycoprotein (P-gp) antibody

Tumor cell multidrug resistance (MDR) is one of the main reasons leading to the failure of chemotherapy. It is currently recognized that the main mechanism of MDR work is the efflux of p-glycoprotein (P-gp). P-gp is an energy-dependent transporter that can transport many compounds with different structures out of the cell in the reverse direction. P-gp is overexpressed in tumor drug-resistant cells, and the P-gp antibody-modified nano-drug carrier system provides a targeted strategy for efficiently capturing such cells. Ma et al. proposed a P-gp antibody-modified porous hydrogel particle for capturing drug-resistant tumor cells, and the results showed that it can adsorb more drug-resistant tumor cells. This strategy gives the hydrogel material the ability to efficiently capture and detect drug-resistant tumor cells.^207^

#### Prostate-specific membrane antigen (PSMA)

PSMA is a transmembrane glycoprotein that is overexpressed on prostate cancer cells and is a recognized biomarker for prostate cancer. Cho et al. developed a multifunctional nanosystem to modify the paclitaxel-loaded composite nanocarrier with anti-prostate-specific membrane antigen (anti-PSMA). The composite nanoparticles were successfully targeted to the LNCaP cells with the targeted receptor PSMA and the tumor area in vivo and in vitro.^208^

#### Ephrin type-a receptor 3 (EPHA3)

EPHA3 is a membrane-associated receptor that is overexpressed in the stroma and vasculature of gliomas, but not in normal tissues.^209^ It is a functional target for the treatment of glioblastoma (GBM). Chu et al. developed EPHA3 tyrosine kinase antibody-modified PLGA NPs for targeting glioblastoma. The uptake of anti-EPHA3 modified NPs in cells was significantly enhanced, and showed a higher distribution in the brain, indicating that anti-EPHA3 has a brain-targeting effect.^210^

#### Glypican-3 (GPC3) antibody

GPC3 belongs to the heparan sulfate proteoglycan family. It is a cell surface glycoprotein in which heparan sulfate glycosaminoglycan chains are covalently linked to the protein core. GPC3 is overexpressed in Hepatocellular carcinoma (HCC) tissues, but not in the liver of healthy adults. This specificity makes GPC3 suitable as a tumor antigen for targeted therapy.^211^ Many anti-GPC3 monoclonal antibodies target the GPC3 molecule that recognizes the HCC cell membrane structure, leading to antibody-mediated endocytosis. Tang et al. developed an Anti-GPC3 Monoclonal antibody (mAb) (Clone 9C2) modified sorafenib-loaded polymer nanoparticles (NPs). The GPC3 molecule on the surface of HepG2 cells can mediate the endocytosis of NPs, so that the cells can more effectively enrich mAb-modified NPs and exert a higher tumor suppressor effect.^212^

#### Epithelial cell adhesion molecule (EpCAM) antibody

EpCAM is a highly expressed marker in cancer, especially in circulating tumor cells (CTC), and is closely related to poor prognosis.^212^ Therefore, it is an interesting strategy to capture CTC based on anti-EpCAM antibody-modified nanomedicine. Li et al. used Anti-EpCAM antibody to modify reduced graphene oxide (rGO) films, which showed extreme sensitivity to CTC, and could efficiently capture CTC from fresh whole blood, with high specificity and low background.^213^ In addition to the above specific ligand modifications, a variety of tumor cell surface markers such as CD44, CD47, CD20, CD147, CD31 have been identified as effective targets for specific and selective anti-tumor therapy.^214–216^ These proteins are involved in tumor proliferation, invasion and metastasis, and play a pivotal role in tumor occurrence and development. Numerous studies have used their corresponding antibodies to modify the nano-drug carrier system to target-specific tumor cells or vascular endothelial cells, so that the drugs can be more accumulated and taken up, and they can play a more effective anti-tumor effect.

### Peptide modification

In the process of tumor occurrence and development, many important molecular targets have been discovered, and the development of specific diagnostic and therapeutic drugs targeting tumors has attracted much attention. Peptide is a tumor-specific ligand, usually composed of less than 50 amino acids. It has small size, high affinity, good stability, easy modification and low immunogenicity.^217^ It has attracted more and more attention in the field of tumor diagnosis and treatment. We summarized the peptides currently used to modify nanomedicine (Table 1), and introduced the main peptides in detail.

**Table 1:** Representative peptide modifications of smart nanoparticles for cancer therapy

Peptide type	Ligand	Peptide sequence	Target receptor	Nanocarrier	Tumor type	Ref.
Targeting peptide	RGD	Arg-Gly-Asp	αvβ3/αvβ5	Bisulfite-zincII-dipicolylamine and ultrasmall Au-ICG nanoparticles	GBM	^218^
	C16Y-L	DFKLFAVYIKYR-GGC	αvβ3	Liposomes	colon26 tumor	^220^
	NLS	GGGPKKKRKVGG	Nuclear Protein(nuclear localization)	Gold nanospheres (Au NPs)	Human oral squamous cell Carcinoma(HSC-3)	^350^
	Angiopep-2	TFFYGGSRGKRNNFKTEEY	Low-density Lipoprotein receptors (LPRs)	Red blood cell membrane	GBM	^221^
	Her2	YCDGFYACYMDV	Human epidermal growth factor receptor 2	Hyaluronic acid (HA)-based micelles	Breast cancers	^225^
	IL-13	VDKLLLHLKKLFREGQFNRN FESIIICRDRTC	IL-13 receptor	Mesoporous silica (MSN)	GBM	^226^
	G11	YHWYGYTPQNVI-GGGGC	Epidermal growth factor receptor (EGFR)	PEG-PLGA diblock copolymers	Human ovarian adenocarcinoma (SKOV3)	^224^
	T7	HAIYPRH	Transferrin receptor (TfR)	Liposomes/siRNA NPs	MCF-7 breast cancer	^228^
	DUP1 peptide	CFRPNRAQDYNT	Prostate-specific membrane antigen (PSMA)	Polymeric micelles	Prostate tumor	^351^
	G12	DHLASLWWGTEL	Glypican-3	Liposomes	Hepatocellular carcinoma (HCC)	^352^
	NGR peptide	Asp-Gly-Arg	CD13	PEG-CdSe/ZnS QDs	Glioma	^353^
	RVG peptide	YTIWMPENPRPGTPCDIFTNSRGKRASNG	Nicotinic acetylcholine receptor	Poly(D,L-lactide-co-glycolide) (PLG) nanoparticles	Neuroblastoma	^354^
Cell-penetrating peptide	TAT	YGRKKRRQRRR	CPPs	MSN	C6 rat glioma (C6) cells	^231^
	TD	ACSSSPSKHCG	CPPs	Liposomes	Melanoma	^232^
	Octaarginine (R8)	RRRRRRRR	CPPs	Lipoplex	Colorectal carcinoma	^233^
Therapeutic peptide	Tat-POSH-C	Ac-GRKKRRQRRRPP-RPRKEDELELRKGEMFLVFER-C	–	Micelle	Non-Hodgkin lymphoma	^234^

#### RGD peptide

The α_v_β_3_ integrin receptor plays an important role in signal transduction. The receptor is highly expressed on the surface of tumor cells and activated endothelial cells and new blood vessels in tumors, and has been proven to be an effective target for tumor therapy. RGD peptides have high affinity with αvβ3 integrin receptors, which makes RGD a unique molecular ligand targeting tumor. The use of RGD peptides to modify nanocarriers is currently the most extensive peptide modification. The basic sequence of RGD peptides is arginine-glycine-aspartic acid (Arg-Gly-Asp). Peptides used as nano-modifications also include cRGD, c(RGDyK), c(RGDyC, c(RGDfK), RGDfC, RGDC, RGD-N3. Gao et al. used RGD polypeptide to modify bisulfite-zinc^II-^dipicolylamine (Bis(DPA-Zn)-RGD). Due to the new blood vessel targeting properties of RGD, Bis(DPA-Zn)-RGD can be selectively delivered to tumor sites. In the tumor environment, it is assembled with ultra-small Au-ICG nanoparticles to form R/Au-ICG nanoclusters in situ. The newly developed gold nanoclusters can penetrate the blood-brain barrier and use its high photothermal effect to inhibit the growth of brain tumors in mice.^218^ Liang et al. used cyclic Arg-Gly-Asp acid (c(RGDyC)) peptides to modify fluorescent gold nanoclusters for cancer radiotherapy. Based on the targeting properties of c(RGDyC) to αvβ3 integrin-positive cancer cells and good biocompatibility, it avoids serious damage to surrounding normal tissues caused by enhanced radiotherapy.^219^ In sum, the modification of RGD peptides is mainly to enhance the tumor targeting of the payloads.

#### C16Y peptide

C16Y peptide is also a targeting peptide of α_v_β_3_ integrin. Negishi et al. used C16Y peptide to modify liposomes (C16Y-L) and tested its targeting ability in vivo and in vitro analysis. Its cellular uptake in colon 26 cells is higher than that of liposomes, and it can specifically attach to tumor slices. In vivo studies have found that C16Y-L not only accumulates in tumor tissues, but also in tumor blood vessels. This proves that C16Y peptide has specific tumor targeting.^220^

#### Angiopep-2 peptide

Angiopep-2 is also a targeting peptide. Angiopep-2 has high affinity with low-density lipoprotein receptors (LPRs) overexpressed on the surface of blood-brain barrier endothelial cells and GBM cells, and is considered a target effective ligand for glioma. Liu et al. used Angiopep-2 peptides to modify the red blood cell membrane as the outer shell, and citraconic anhydride grafted poly-L-lysine (PLL-CA) as the intermediate layer for siRNA delivery (Ang-RBCm-CA/siRNA). This functionalized nanocomposite can penetrate through the blood-brain barrier and enhance the accumulation and retention of SiRNA in tumors. Provides an effective and multifunctional platform for GBM targeted gene therapy.^221^

#### A54 peptide

In a phage display random peptide library, the A54 peptide is a peptide that binds to hepatic carcinoma. For the human hepatoma cancer cell line BEL-7402, it is the most effective peptide. Therefore, the A54 peptide is used as a target ligand for hepatic cancer. Zhang et al. have synthesized A54-TPGS (Polyethylene glycol 1000 vitamin E succinate) through esterification, and combined with calcium phosphate nanoparticles to form a multifunctional drug-carrying system, which has higher tumors tissue homing characteristics and longer tumor tissue residence time.^222^ Du et al. developed a PEGylated stearic acid grafted chitosan micelle (PEG-CS-SA), and A54 peptide was used as a targeting ligand to functionalize the PEG-CS-SA micelle. It exhibits special internalization ability for liver cancer cells in vitro, and has a high distribution ability in liver and liver cancer tissues in vivo. It can more effectively inhibit tumor growth and reduce toxicity.^223^

#### EGFR peptide

EGFR is overexpressed in a variety of highly aggressive tumors and is a promising target for tumor therapy. The modification of targeting ligands for EGFR has attracted great attention. Chernenko et al. designed an EGFR targeting ligand peptide, termed G11, to modify PEG-PLGA di-block copolymers. Compared with the cellular uptake of nontargeted nanoparticles, EGFR polypeptide-modified nanoparticles are rapidly internalized in ovarian cancer cells, leading to significant intracellular accumulation.^224^

HER2 is a useful target for the therapy of breast and ovarian cancer, as was already mentioned. The only HER2 targeted medication for the treatment of breast cancer that has received FDA approval is HER2 cyclic peptide. It can specifically recognize low concentrations of HER2 receptors, so HER2 peptides can be used as tumor cell-specific peptides ligand. Chen et al. prepared a hyaluronic acid (HA)-based pH-responsive mixture micelle, modified with Her2 peptide, which can reach the tumor site more effectively and has good tumor killing activity.^225^

#### Interleukin-13 (IL-13) peptide

IL-13 is a cytokine that binds to two receptor chains. It is highly expressed in a variety of malignant tumors, but the expression level in normal tissues is very low or undetectable. IL-13 has been developed as a tumor-specific targeting ligand. As a targeting ligand, proteins such as IL-13 have the disadvantages of large molecular weight, variability, and difficulty in manipulation. IL-13 peptide (IP) is a 32-amino acid peptide that is easy to synthesize and is an effective strategy for targeting IL-13 receptors. For the first time, Wang et al. used IP as a glioma targeting ligand to modify MSN, as a new type of carrier to deliver doxorubicin, which can significantly increase drug uptake by U251 cells and accumulate in the nucleus, enhanced targeting.^226^

#### T7 peptide

Transferrin receptor (TfR) is highly expressed on the surface of cancer cells. Therefore, the transferrin receptor is also the most common target receptor for drug delivery systems. T7 peptide is a heptapeptide with high binding affinity to TfR. T7 peptide modified smart drug carrier has attracted widespread attention in the field of drug delivery. Zhang et al. used T7 peptide modified liposomes as a carrier for transporting HER2 inhibitors, which can actively target breast cancer tumors and reduce the toxicity to normal tissues.^227^ Gao et al. also confirmed that T7 peptide-modified polymers exhibited higher cell uptake efficiency and rapid endosomal/lysosome escape ability in breast cancer MCF-7. Targeted delivery mediated by transferrin receptor produces the greatest tumor suppressor effect in vivo.^228^

#### APRPG peptide

Angiogenesis is closely related to tumor growth and metastasis, and targeted angiogenesis has shown significant anti-tumor efficiency in cancer treatment. Therefore, the research of targeted angiogenesis drugs has attracted much attention. Wang et al. prepared APRPG polypeptide modified poly(ethylene glycol) polylactic acid (PEG-PLA) nanoparticles to encapsulate angiogenesis inhibitors, using APRPG peptides to specifically target the over-expressed α_v_β_3_ integrin during tumor angiogenesis, and more conducive to the effective load to inhibit tumor angiogenesis.^229^

More targeting peptides are introduced in Table 1. In addition, the modification of cell-penetrating peptides is another category in smart nano-delivery systems. Cell-penetrating peptides (CPPs) are cationic short peptides with specific conserved sequences, which have the ability to transport payload into cells.^230^ Next, we introduce several cell penetrating peptides.

#### TAT peptide

TAT peptide is the most widely used CPP in drug delivery. The TAT peptide is derived from the protein transduction domain of the human immunodeficiency virus Tat protein. The 11 amino acids YGRKKRRQRRR of Tat is the smallest sequence that leads to cell penetration. TAT can not only penetrate cell membranes efficiently, but can also carry macromolecular drugs that are 100 times larger than Tat across cell membranes. This feature has potential applications in the delivery of large molecules such as peptide or protein drugs. Kanazawa et al. used TAT peptides to modify mesoporous silica nanoparticles to form a multi-layer nanocomposite for the co-delivery of doxorubicin and siRNA. The modification of the TAT peptide gives the drug the ability to penetrate various biological barriers, thereby effectively and selectively delivering siRNA and DOX to the cytoplasm and nucleus, respectively.^231^

#### TD peptide

TD is a biologically stimulating peptide that is used to enhance skin penetration. Compared with cell penetrating peptides that promote the binding of drugs through the stratum corneum into living skin cells and keep the drugs in the skin, TD has the advantage that it can temporarily open the paracellular pathway and promote the drug to pass through the skin completely. Zou et al. prepared TD-modified vemurafenib-loaded liposomes (Vem-TD-Lip). After modification, the penetration amount of Vem in the cell was significantly increased. This shows that TD peptide promotes the delivery of Vem-TD-Lip on the skin.^232^

#### Octaarginine (R8) peptide

R8 is a cationic peptide containing basic amino acid residues. It is a promising cell penetrating peptide, which can promote transportation across the absorption barrier by forming an electrostatic bond with the negatively charged part of the cell membrane. Singh et al. synthesized an octaarginine (RRRRRRRR)-oxaliplatin conjugate through a specific heterobifunctional linker, which can quickly and successfully deliver oxaliplatin to colon cancer cells, showing quite high resistance tumor activity.^233^

In addition to targeting peptides and cell penetrating peptides, some biologically active peptides used as therapeutic peptides also provide exciting alternatives for tumor treatment. Smith et al. lipidated bioactive peptides modified with cell penetrating peptides (Tat-POSH-C), and then they self-assembled into polypeptide amphiphilic micelles (PAMs) driven by hydrophobicity, that is, the therapeutic peptides themselves assemble into nanogels bundle. Finally, the C10.36 aptamer was loaded on the surface of the micelle. This therapeutic peptide strategy induced significant peptide toxicity in human lymphoma cells.^234^

Compared with single-peptide modification, double-peptide modification can endow smart nanocarriers with more functions, which has also aroused great interest. RGD-N_3_ (cyclo (Arg-Gly-Asp-d-Phe-Lys(Azide))) can target tumor’s αvβ3 integrin receptor, Beclin 1 derived peptide is a functional peptide that can bind to Class III phosphatidylinositol 3-kinase (PI3KCIII)/Vps34, initiate cancer cell autophagy. Zhou et al. prepared melanin-like polydopamine nanoparticles modified with RGD-N3 and Beclin 1 double peptide for the treatment of breast cancer, which significantly promoted the autophagy activity of cancer cells.^235^ Yang et al. used three peptides to modify gold nanoparticles (GNP). The targeting peptide RGD promotes the absorption of NP on the cell surface. The cell penetrating peptide NLS has a nuclear localization signal and promotes nuclear transmission. The third Peptides (pentapeptides) cover the surface of GNP, protect GNP from being bound by serum proteins, and also stabilize the GNP complex. The modification of the tripeptide showed a 5-fold NP uptake and effective nuclear localization.^236^

### Aptamer modification

Nucleic acid aptamers are single-stranded oligonucleotides (ssDNA or ssRNA), which can be folded into a unique tertiary structure to identify its target.^237^ Since 1990, Tuerk’s and Szostak’s groups firstly selected highly specific aptamers through a process called “systematic evolution of ligand by exponential enrichment” (SELEX).^238,239^ Research on aptamer-mediated targeted drug delivery systems has caused A strong interest. At present, high-affinity aptamers recognize more than 900 different targets, including cytokines, growth factors, proteases, immunoglobulins, cell surface receptors, and cell adhesion molecules.^240,241^

The targeting mode of the aptamer is similar to that of antibody modification, but the aptamer has attractive advantages: 1) Low immunogenicity: the aptamer has no Fc region, which avoids the interaction with immune cells or other specific cells; 2) Small molecular weight: The molecular weight of the aptamer is usually between 6 and 30 kDa, which is much smaller than the molecular weight of the antibody (about 150 kDa), which is more conducive to penetration into the deep layer of the tumor; 3) aptamer production does not need any biological system, and it is easier to carry out large-scale production with low inter batch variation; 4) High stability: aptamers have higher stability than proteins in biological fluids, and can be denatured and re-denatured many times without losing activity.^242^ It can be seen that aptamers are a less restrictive alternative to antibodies with high affinity to the target and have great potential in cancer treatment. Various promising nucleic acid aptamer nanomedicine systems have been reported for drug therapy, gene therapy and tumor imaging. We will introduce recent developments in aptamers used in targeted drug delivery systems.

#### PSMA aptamer

The development and application of PSMA-specific aptamers provide a potential strategy for the diagnosis and treatment of prostate cancer. A9 and A10 aptamers are PSMA aptamers screened by Lupold et al. Farokhzad et al. published the first report of targeted drug delivery with nanoparticle-aptamer bioconjugates.^243^ They used PSMA’s RNA aptamer A10 and polymer nanoparticles to form bioconjugates, and proved that these bioconjugates can effectively target prostate cancer epithelial cells and be more absorbed. Compared with A10, the second-generation RNA aptamer A10-3.2 has a higher binding efficiency with PSMA.^244^ A9 aptamers have also been used in various functional studies and have been modified on a variety of nanocarriers including gold nanoparticles and liposomes for targeted delivery of therapeutic drugs or genes.^245^ In order to obtain a shorter RNA aptamer, William M. Rockey et al. truncated the A9 aptamer to obtain the A9g aptamer, which improved the stability of the aptamer and reduced the difficulty of synthesis, while retaining the high-affinity binding ability to PSMA.^246^

However, PSMA is only overexpressed in androgen-independent prostate cancer and cannot specifically treat PSMA(−) cell lines. In order to treat prostate cancer more completely, it is important to target both PSMA(+) and PSMA(−) cell lines at the same time. The modification strategy of dual aptamers provides a good breakthrough to solve this problem. A peptide aptamer (DUP-1) has been identified as an aptamer for PSMA(−).^247^ Hunho Jo’s team simultaneously conjugated the A10 aptamer and DUP-1 aptamer to the PEGylated gold nanostars (Dual-AuNS) via disulfide bonds. Studies have shown that Dual-AuNS can simultaneously target PSMA(+) and PSMA(−) cells, and has high selectivity.^248^ Coincidentally, Jing et al. also prepared a dual aptamer modified (the second-generation RNA aptamer A10-3.2 and DUP-1) delivery system to deliver tumor suppressor genes and doxorubicin. This system enhances drug uptake and gene expression in prostate cancer cells.^249^

In addition to the above PSMA aptamers, more aptamers have also been found and synthesized to target the treatment of prostate cancer by coupling with nanoparticles.^245^

#### Mucin 1 (MUC1) aptamer

MUC1 is a mucin glycoprotein overexpressed on some adenocarcinoma cells and is considered an important tumor surface marker, especially breast cancer. It has been used as a potential target for the treatment of breast cancer.^250,251^ Therefore, MUC1 aptamers, which have high specific recognition ability with cancer cells overexpressing MUC1, are promising delivery agents for the development of targeted nanoparticles. DNA nanocage (DNA polyhedral nanostructure) is a nano-delivery system based on endogenous biomolecules. Han et al. used complementary base pairing to modify the MUC1 aptamer into tetrahedral DNA nanocages (Td) for self-assembly to load doxorubicin. The complex reduces the uptake rate in normal cells and improves the uptake efficiency in breast cancer cells. This high specificity reduces systemic toxicity and has an effective tumor suppressor effect.^252^

#### AS1411 aptamer

AS1411 aptamer is a 26-molecule DNA oligonucleotide accidentally discovered by Bates et al.^253^ It can specifically bind to nucleolin, which is highly expressed on the nucleus and on the surface of tumor cell membranes.^254^ He et al. used AS1411 aptamers to modify the multiarmed amphiphilic cyclodextrins (CDEH) self-assembly delivery platform for protein delivery. This nanocarrier can preferentially accumulate in tumors and effectively inhibit tumor growth.^255^ Liang et al. also used AS1411 aptamers to modify micelles to improve their targeting functions. The system simultaneously delivers chemotherapeutics and genes to overcome the multidrug resistance of tumors.^256^

#### Anti-EGFR aptamer

The specific ligands used to modify the nanosystem targeting the EGFR targets, in addition to the antibodies and peptides described above, EGFR aptamers have been successfully used to recognize EGFR-expressing cells.^257^ Li et al. used anti-EGFR aptamers combined with chitosan and anchored into liposomes to co-deliver erlotinib and PFOB, which can reverse hypoxia-induced drug resistance. This liposome complex can specifically bind to non-small cell lung cancer overexpressing EGFR, and effectively deliver the drug to the targeted location.^258^

#### Protein tyrosine kinase (PTK) 7 aptamer

PTK7 is a transmembrane receptor that is upregulated in many common cancers including acute T-lymphocytic leukemia (T-ALL) and is a potential biomarker of T-ALL.^259^ Sgc8 can specifically identify leukemia cells (CEM) by recognizing PTK-7. Targeted delivery using Sgc8 aptamer modification has attracted a lot of research in the field of intelligent drug delivery. Jin et al. used Sgc8 aptamers to modify biocompatible DNA nanostructures to load the photosensitizer methylene blue.^260^ CEM cells show specificity and high uptake of aptamer modified drug delivery system, which can induce stronger antitumor activity in photodynamic therapy.

The application of aptamers has attracted more extensive research, and more smart targeted delivery systems modified by aptamers for cancer surface receptors have been developed. For example, the aptamer AptHER2 for HER2 receptor, the aptamer AraHH001 for tumor endothelial cells, the aptamer for glucose transporters (GLUT-1).^261,262^

### Other targeting ligand

#### Folic acid modification

The folate receptor (FR) is overexpressed on the surface of a variety of cancers, including breast, kidney, colorectal, brain, and ovarian cancers.^263–265^ Folic acid can highly specifically bind to FR, which promote the internalization of folate-targeted NPs into the cytoplasm through receptor-mediated endocytosis. The acidic microenvironment of the cytoplasm causes the separation of FR and folic acid-coupled NPs, and the NPs were released into the cytoplasm to exert anti-tumor effects.^266^ Therefore, the targeted therapy of folic acid-modified nanoparticles for tumors is a hot spot in the field of smart nanoparticles. Besides, in ligand-modified active targeted drug delivery systems, compared with antibodies, folic acid has the following characteristics: 1) higher tumor permeability; 2) higher self-stability, including in acidic, alkaline and solvent ; 3) Relatively low toxicity; 4) Highly economical.^266^ A large number of smart drug delivery systems based on folic acid modification have been developed. Cui et al. coupled folic acid to amphiphilic chitosan and wrapped it on the surface of upconversion nanoparticles (UCNPs), and then connected it with the photosensitizer ZnPc through hydrophobic interaction to construct a multifunctional nanosystem. The multifunctional nanostructures have imaging functions and can visually observe their selective aggregation in tumor cells overexpressing folate receptors. In deep tumors, more reactive oxygen species are generated after excitation by 980 nm near-infrared light. Compared with conventional visible light-activated PDT, it has a more significant tumor inhibitory effect.^267^ The folic acid-modified nanoparticles constructed by Luiz et al. also showed 3.6 times higher cellular uptake than unmodified nanoparticles.^263^ These results prove that folic acid-modified nanoparticles are an excellent choice in the field of smart nano-delivery.

#### Transferrin modification

Transferrin receptor (TfR) is overexpressed on a variety of metastatic and drug-resistant tumor cells (including brain cells). Therefore, it can be used as a target receptor to recognize tumor cells, and transferrin receptor has been widely used in targeting strategies. Transferrin (Tf) can specifically recognize TfR and can be used as a targeting moiety to be coupled to a delivery system to achieve targeted drug delivery. Current drug delivery systems based on Tf modification can achieve selective cellular uptake, cross the blood-brain barrier, limit systemic toxicity, and reverse multidrug resistance.^268^

In brain tumors including gliomas, transferrin receptors are highly overexpressed on the surface of brain capillary endothelial cells and tumor cells. The transferrin receptor targeting ligand has the ability to cross the blood-brain barrier, which is currently the main obstacle to the treatment of many brain diseases including glioma. Receptor-mediated endocytosis and endocytosis are the main ways for nanoparticles to cross the blood-brain barrier to reach gliomas.^269^ Liu et al. prepared transferrin (Tf) modified magnetic nanoparticles for the delivery of siRNA (siPLK1). The uptake of siPLK1 by U87 cells can significantly increase, improve the BBB penetration efficiency of siPLK1 in vivo, and can selectively accumulate in the brain tissue, thereby inhibiting the growth of glioblastoma tumors and prolonging the survival period.^270^ The most important feature of transferrin is that it can transport iron, which is an important cell growth regulator. Studies have found that artesunate (AS) can be activated by iron in cells and exert strong cytotoxicity to tumor cells in vivo and in vitro. Therefore, Hou et al. anchored Tf on the surface of copper sulfide nanoparticles as a targeting molecule, loaded with artesunate. The system can specifically target tumor cells and be absorbed by breast cancer cells through tf-mediated endocytosis, while delivering As and iron ions to the tumor, thereby enhancing anti-tumor activity.^271^

More studies have also proved that transferrin-coupled or covalently linked nanoparticles endow the NPs function of targeting tumors, which can more effectively realize the active targeting of drugs and achieve the purpose of tumor diagnosis or treatment.^272–275^

## Payload

The ultimate goal of the development of smart nanoparticles is that the payloads can better serve patients: enhance curative effect, reduce toxicity and prolong survival.^276^ In order to achieve these goals, researchers and medical experts have established various methods to overcome the inherent defects of current drugs. With the development of modern biomedicine, the type of payloads that can be carried by smart nanoparticles is also becoming more diverse, including small molecule drugs, nucleic acids, peptides, proteins and live cells, etc.

Small molecule medications were primarily the therapeutic payload of nanoparticles decades ago. To improve drug solubility and bioavailability, control drug release, optimize activity and adjust pharmacokinetic properties are the initial efforts of drug delivery for small molecule.^277^ Later, as new generations of medicines such as proteins and peptides, nucleic acids, and living cells emerged, delivery problems also cropped up, such as the immunogenicity and bioavailability of proteins and peptides, the stability and intracellular transfection efficiency of nucleic acids, and the survival rate and scalability of living cell delivery.^278–280^ Therefore, drug delivery technology also needs to evolve to address emerging demands in advanced cancer treatment.

### Small molecules

Small molecules are chemical drugs with molecular weight less than 900da. Small molecule medications can quickly disperse in biological fluid, pass various biological barriers, and cross cell membranes due to their tiny size features. These benefits make it possible for small molecules to move swiftly through the body’s intricate circulatory system and communicate with practically all tissues and cell types. But because this method is predicated on small molecule medications dissolving readily in biological fluids, the effectiveness of small molecule drugs that are insoluble is likewise constrained.^281^ About 90% of preclinical small molecule candidate drugs have poor solubility, so the realization of this process is very challenging.^282^ Adjusting local microenvironment to improve drug solubility is an important strategy to improve drug bioavailability, especially by changing the structure of small molecules to regulate their physical and chemical properties, so as to improve the dissolution, diffusion or absorption properties.^283^ For example, chemical bridging such as disulfide bond can promote the self-assembly of hydrophobic small molecule prodrugs, so as to improve the absorption and bioavailability of drugs.^284^

Nanoparticle and particle based systems have been used to overcome the problem of drug solubility, enable small molecules to be transported to their action sites, and reduce off target side effects.^285^ Nanoparticle therapy has been approved to be widely used in cancer treatment, vaccination and other indications.^286,287^ Appropriate charge and surface materials have greatly improved the delivery control of nanoparticle-based therapeutic drugs.^288–290^ Previous research revealed that PEG coating is a useful technology for increasing the retention of particles in tumor sites and extending the circulating half-life of particles.^291^ The successful listing of pegylated liposome Doxil, the first nanoparticle therapeutic drug, in 1995 proved the feasibility of this technology.^292^ Since then, substantial preclinical research has been conducted on smart nanoparticles to solve the persistent problems with target-specific delivery.

### Proteins and peptides

Although the research of small molecule drug delivery has established a solid foundation, the target of small molecule drugs accounts for only 2–5% of the human genome, so the development of other alternative therapies is necessary.^293^ Peptides (2–50 amino acids) and proteins (more than 50 amino acids) have better selectivity for human specific targets. The larger molecular size and diverse quaternary structure enhance their interaction with specific protein pockets, making peptides and protein drugs have better biological activity and lower toxicity than small molecules.^294,295^ The complex structure not only improves the activity and selectivity of peptides and proteins, but also increases the instability.^296^ Under conventional storage conditions, peptides and proteins are easy to degrade, and are extremely sensitive to ubiquitin proteasome system, physiological temperature and pH changes in vivo.^297–299^ For example, carnosine drugs that can be used for cancer treatment exist in human blood for less than 2 min, but after self-assembly to form nano drugs, the half-life and anti-cancer ability in vivo are significantly enhanced.^300^

PEG modification has proven to be the most effective method for reducing protein immunogenicity and extending its half-life. PEG can protect immunogenic epitopes and increase a drug’s hydrodynamic diameter, reducing renal clearance and extending the drug’s half-life in the blood.^301^ In a “humanized” scaffold model of the bone marrow, carfilzomib-loaded polymeric micelles (CFZ-PM) based on poly(ethylene glycol)-b-poly(N-2-benzoyloxypropyl methacrylamide) (mPEG-b-p(HPMA-Bz) have been created to increase the maximum tolerable dose of carfilzomib.^302^ Another strategy is to introduce protease inhibitors to interfere with the degradation process of peptides and proteins in biological fluid, and then adjust the microenvironment to achieve the purpose.^303^ Peptides and proteins have clear size restrictions in the process of penetrating biological barriers because of the huge size of the molecule itself.^304^ It goes without saying that this encourages the creation of penetration enhancers, which can control the microenvironment to buffer local stomach pH or actively accelerate the cross-cell absorption of peptides or proteins.^305^ The successful launch of the first oral glucagon like peptide (GLP-1) Rybelsus is the result of the successful application of this strategy.^280^ Controlled release technology for small molecule therapy is also applicable to peptides. For instance, the particulate drug library’s sustained release of the peptide hormone leuprorelin can lower the need for subcutaneous injections, lessen side effects, and be commercially successful (Lupron Depot).^306^ However, a prominent delivery difficulty is the use of stimulus response delivery systems to simulate the natural regulation of peptide and protein secretion by the host, which is particularly important for the use of appropriate therapies to replace natural biological processes.

### Nucleic acids

Peptide and protein drugs have greatly expanded the number of drug targets. However, since nucleic acid medications have a better ability to regulate gene expression, they can be utilized to silence or repair defective genes and promote the production of useful therapeutic genes.^307,308^ Due to their great sensitivity to nuclease degradation, naked nucleic acids have a short half-life.^309^ The human immune system is also eliminating and adapting to exogenous RNA and DNA at the same time. Cell internalization and endosome escape are necessary for the delivery of nucleic acid medications into the cytoplasm (small interfering RNA and mRNA) or nucleus (ASOs, DNA, CRISPR) in order for them to work.^310^ Therefore, chemists have creatively modified nucleic acid bases, sugar rings, 3 ‘and 5’ ends. These modifications are resistant to nuclease degradation, reduce immunogenicity and improve the interaction with target cells.^311^ Environmental control has been demonstrated to enhance the intramolecular targeting of nucleic acids in preclinical experiments. To complete endosomal escape and cytoplasmic distribution, nucleic acid carriers, for instance, can buffer endosomal pH or form lipid interactions with endosomal membranes.^312^ Additionally, endosomal membranes have been damaged or rebuilt using cell penetrating peptides to enhance the intracellular delivery of nucleic acids.^313^

The success of nucleic acid drugs largely benefits from the development of drug delivery system using smart nanoparticles. For example, Onpattro, the first siRNA drug listed in 2019, uses lipid nanoparticles to deliver chemically modified siRNA, which can achieve cell targeting, uptake and endosomal escape.^314^ Early research demonstrated the viability of non-viral vector gene therapy with a primary focus on cationic liposomes and lipid nucleic acid medicines.^315^ Lipid nanoparticles can efficiently load negatively charged nucleic acids, and its surface charge enhances cell uptake and endosomal escape.^316^ PEGylated, neutral, and ionizable lipids are employed as stable carriers in lipid delivery systems designed specifically for siRNA, despite toxicity, complement activation, and poor biological distribution also encouraging their development.^317,318^ Because they maintain the benefits of high loading efficiency and endosomal escape of liposomes, ionizable cationic lipids are particularly significant. They can also increase the transport efficiency of siRNA by lowering toxicity and enhancing biological dispersion.^319^ At present, lipid nanoparticles are also widely used in the delivery of mRNA and other nucleic acid drugs.^314^ Hyaluronic acid has been attached to lipid nanoparticles by Cohen et al. for the treatment of gliomas. Local convection-enhanced delivery (CED) of the hyaluronic acid functionalized lipid nanoparticles dramatically increased lifespan in the U87-bearing mouse model. These smart nanoparticles were employed to deliver siRNA against PLK1, which is involved in the malignant transformation of glioma cells.^279^ In general, smart nanoparticles can enhance the targeting and stability of pharmaceuticals. The interaction between medications and molecules, cells, and tissues in the human body can be better managed using package modification techniques that incorporate microenvironment modification elements made possible by smart nanoparticles.

### Living cells

Natural cell processes are used by living cells to control or carry out important biological activities. For instance, reprogrammed immune cells can use the immune system to treat cancer and administer vaccinations, while microorganisms can interact with the microbiome to control metabolic processes, chronic inflammatory processes, and mucosal immunity.^320–322^ Additionally, living cells are modifiable. The most well-known instance is CAR (chimeric antigen receptor) T-cells. They are genetically modified cytotoxic T cells that target particular cancer-related antigens and were given clinical approval in 2017.^323^ In reality, CAR T-cell treatment emphasizes the benefits and capabilities of cell therapy: the innate capacity to target the illness site, the potent activity of the action site, the capacity to engage the immune system directly, and the capacity to multiply in vivo.^324^ Other FDA approved adoptive cell therapies are sipuleucel-T (Provenge, used to treat prostate cancer) and umbilical cord blood stem cells.^325^ Cells can also be designed to secrete drugs or catalyze key biological reactions, so they can be used as warehouses for drug factories.^326^ In order to avoid these issues, Matthias et al. have created a technique to quickly program circulating T cells with tumor-recognition abilities. They specifically show how leukemia-targeting CAR genes may be successfully inserted into T-cell nuclei by DNA-carrying smart nanoparticles, resulting in a long-lasting disease remission.^327^

The delivery of living cells also has its own unique challenges. Cells need a far higher dose of therapeutic medications than any other kind, so they might be swiftly trapped in lung capillaries and removed.^328^ Low permeability of cells in solid tumors is a problem for adoptive cell therapy because of the size of living cells and the unfavorable tumor microenvironment. This limits their current clinical application in hematological malignancies.^329^ Additionally, the environment and host of supplied live cells play a significant role in the survivability, durability, and maintenance of beneficial cell phenotypes.^330^ There are also practical problems related to the large-scale production of therapeutic living cells.

With the rapid development of nanotechnology, researchers began to have new strategies to solve these challenges, including designing effective smart nanoparticles to improve adoptive cell therapy for cancer treatment. Engineering therapeutic cells for in vivo imaging, enhancing tumor infiltration and achieving functional sustainability in vivo, and producing tumor-killing T cells in vivo are all possible uses for multi-scale artificial antigen-presenting cells. These cells can also be used for cell proliferation and stimulation in vitro, increasing the transduction efficiency of tumor targeted areas, and all of the above.^331^

### Combination therapy

Single drug therapy based on single chemotherapy regimen is often not ideal in clinical efficacy, the complexity of cancer makes it necessary to develop a combination of two or more treatment schemes to achieve better anti-cancer effect. However, how to develop efficient and low toxicity drug combinations to effectively control tumor growth is still a severe challenge. For instance, gemcitabine monotherapy is the standard treatment choice for patients with advanced breast cancer, it does not prolong the median survival time of patients with metastatic breast cancer. Samir et al. have developed a combination of gemcitabine-imiquimod based on hyaluronic acid to stimulate the anticancer activity of immune cells.^278^ This study fully proves that imiquimod can enhance the therapeutic effect of gemcitabine by activating immune cells using smart nanoparticles.

There are many potential benefits of using smart nanoparticles for ratio-metric delivery of synergistic drug combination for cancer treatment in clinical applications.^332,333^ For instance, ratiometric medication combinations supplied by free solution can have their pharmacokinetics and biodistribution altered by smart nanoparticles-based synergistic drug combination delivery.^334^ Traditional free drug combinations cannot reach the tumor after intravenous injection and fail to maintain the correct drug ratio. Additionally, to combat cancer’s multidrug resistance, smart nanoparticles transported synergistic drug combinations into cancer cells.^335^ By using the ERP effect or receptor-mediated extravasation, the smart nanoparticles build up in the tumor, bind to tumor receptors, enter cancer cells by endocytosis, and release medications there.^336^ The released medications exert synergistic activity by moving into the nucleus, which is the location of drug action in this situation. This results in more DNA double strand breaks.^337^

## Artificial intelligence-powered nanoparticles

Artificial intelligence (AI), a branch of computer science, aims to perform complex tasks that require “human intelligence” through computers or computer-controlled machines. There are various subfields of AI, such as machine learning (ML), artificial neural networks (ANN) and deep learning (DL).^338^ In recent years, AI-powered nanoparticles have emerged as a promising approach for cancer therapy, offering potential advances in precision medicine. Combining these nanoparticles with AI techniques has the potential to revolutionize cancer drug delivery and design, early cancer screening and diagnosis, and predicting drug dosage and efficacy.

Drug delivery is one of the primary applications of AI-powered nanoparticles. Firstly, based on omics and nanosensor technologies, accurate biomarker analysis of patient’s tumor is performed to identify suitable patients for clinically targeted drug formulations. Secondly, by implementing AI algorithms, these nanoparticles can be designed to navigate intelligently through the complex tumor microenvironment, selectively target cancer cells, and deliver therapeutic payloads with increased precision. Dahlman et al. designed and optimized a high-throughput DNA barcoding system for the detection of lipid nanoparticles carrying specific nucleic acid barcodes in normal or tumor tissues in vivo, which facilitates the discovery of nanoparticles targeting tumor tissues and cells.^339^ Large datasets can be analyzed and processed by AI algorithms, enabling nanoparticles to dynamically adapt and respond to the specific requirements of individual patients.^340^ This personalized approach has the potential to improve treatment outcomes and reduce adverse effects. In addition, AI-driven drug delivery can act as a “navigator” to remotely transport therapeutic agents in addition to targeting lesion location. An interesting study combines unmanned aerial vehicle (UAV) technology and microneedle patches to build a UAV-mediated targeted drug delivery emergency system that not only identify the position of patients and reach them immediately but also achieve autonomous drug administration to relieve symptoms.^341^

AI algorithms can also aid in the design and optimization of intelligent nanoparticles to surmount the limitations of conventional drug delivery systems and improve the efficacy of cancer treatment. AI can optimize multiple aspects of nanoparticle design, including nanoparticles’ size and charge, drug encapsulation efficiency, interactions with biological membranes, vasculature, biological fluids, and drug release kinetics through machine-learning algorithms and computational models.^342^ AI can accelerate the discovery of novel nanoparticle formulations, predict their behavior in the body, and optimize their properties for specific therapeutic applications. A recent work applied ferritin nanocages loaded with drugs and tracers to 32 different tumor models. Subsequently, data analysis of >67,000 individual blood vessels was performed by image-segmentation-based machine learning (nano-ISML) techniques to predict nanoparticle permeability in the tumor vasculatures. And based on the rational design of nano-ISML-assisted nanomedicines, genetically tailored protein nanoparticles were developed to improve transendothelial transport in low-permeability tumors and assists nanoparticles to enter solid tumors.^343^

Moreover, nanoparticles propelled by AI can enhance the efficacy of therapeutics through real-time monitoring and feedback. By integrating sensors and imaging agents into the nanoparticle system, artificial intelligence algorithms can monitor the response of tumors to treatment, providing valuable insights into therapeutic efficacy and facilitating adaptive treatment strategies. This closed-loop feedback system can maximize the therapeutic response and overcome drug resistance by optimizing drug delivery, adjusting dosages, and even switching therapeutic modalities as required. The combination of AI and nanotechnology can also be used for early cancer screening and diagnosis to reduce the mortality rate of cancer patients. Kim et al. constructed a nanosensor array technology to identify disease spectral fingerprints and combined it with machine learning algorithms to successfully distinguish between ovarian cancer patients and healthy individuals, which can be used to predict ovarian cancer with high sensitivity and specificity.^344^

It is essential to note, however, that the application of AI-powered nanoparticles in cancer therapy is still in its infancy, and several obstacles must be overcome. Among these are assuring the safety and biocompatibility of the nanoparticles, addressing regulatory concerns, and validating the clinical efficacy through rigorous studies and trials.

## Clinical setting

To facilitate the successful translation of smart nanoparticles into clinical applications, thorough assessment of their safety and toxicity is imperative. Here is additional information highlighting the importance of safety considerations in advancing smart nanoparticles towards clinical use:

1. Preclinical evaluation: Prior to clinical trials, extensive preclinical evaluations are necessary to investigate the safety profile of smart nanoparticles. This involves in vitro and in vivo studies to assess their biocompatibility, potential toxicity, and any adverse effects on cells, tissues, and organs.
2. Systemic toxicity: Smart nanoparticles should undergo rigorous evaluation to determine their systemic toxicity. This includes examining their distribution in the body, potential accumulation in organs, and any potential long-term effects on physiological functions. It is crucial to ensure that the nanoparticles do not induce systemic toxicity or harm the overall health of the patient.
3. Immunotoxicity assessment: The immune response triggered by smart nanoparticles is a critical aspect to consider. Immunotoxicity studies are essential to evaluate any immune reactions, including inflammation or immunosuppression, caused by the nanoparticles. Understanding the immune implications helps determine the nanoparticles’ compatibility with the immune system and their potential for immunomodulatory applications.
4. Targeted toxicity: Smart nanoparticles designed for targeted drug delivery or specific therapeutic purposes should undergo evaluations to assess their toxicity at the target site. This involves investigating if the nanoparticles induce any cytotoxicity or unwanted effects in the vicinity of the target area. Ensuring localized safety is crucial for successful clinical applications.
5. Pharmacokinetics and biodistribution: Understanding the pharmacokinetics (absorption, distribution, metabolism, and excretion) and biodistribution of smart nanoparticles is essential for predicting their behavior in the human body. This information helps assess potential accumulation in critical organs, elimination pathways, and the overall clearance of the nanoparticles, minimizing the risk of toxicity and ensuring safe clinical use.
6. Long-term safety: Long-term safety assessments are vital for the clinical translation of smart nanoparticles. These evaluations involve prolonged exposure studies to monitor any chronic toxicity, including the nanoparticles’ potential to induce tumorigenic effects or chronic inflammation. Such investigations provide crucial insights into the nanoparticles’ safety over extended periods.
7. Regulatory compliance: Compliance with regulatory guidelines is paramount to facilitate the clinical translation of smart nanoparticles. Regulatory authorities, such as the FDA, require comprehensive safety data to support the approval of nanoparticle-based therapies. Meeting the regulatory standards ensures that the nanoparticles are safe for human use and paves the way for their successful clinical implementation.

By addressing these safety considerations, researchers and clinicians can establish a solid foundation of evidence regarding the safety and toxicity of smart nanoparticles. This information not only promotes the responsible development of these innovative technologies but also instills confidence in their potential for clinical applications.

**Fig. 5:**
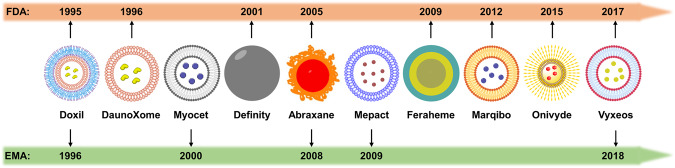
Timeline of the development of smart nanoparticles for cancer diagnosis and treatment

Several clinically approved nanoparticle formulations either by the FDA in the United States or the European Medicines Agency (EMA) in the European Union are used to treat a variety of cancers at different stages (Table 2). The timeline and basic structure of smart nanoparticles for cancer diagnosis and treatment in the clinical setting are shown in Fig. 5. Doxil, a PEG functionalized liposomal doxorubicin, was the first nanomedicine that has been approved for anticancer medications (FDA in 1995).^292^ Rapid clearance is a ubiquitous challenge for nanoparticle-based drug delivery. To increase circulation time, chemical modification with a variety of compounds, including PEG and its derivatives can be utilized to alter the surface of a nanoparticle. Except for Doxil and Onivyde, most of these formulations are non-PEG.^345^ CPX-351 (Vyxeos) is a gel-phase bilamellar liposome nanoparticle with a mean diameter of 107 nm and a strong negative surface potential, which is based on the principle of ‘ratiometric’ dosing of the cytarabine/daunorubicin combination with a molar ratio of 5:1 provides the greatest synergistic effect with the lowest antagonism in vitro and in vivo.^346^ This liposomal nanoparticles are approved from US FDA for the for the treatment of adults with newly diagnosed acute myeloid leukemia (AML) with myelodysplasia-related changes (AML-MRC) and therapy-related AML (t-AML).^347^ Non-liposomal nanoparticle systems that have been approved for cancer treatment include Abraxane and NBTXR3 Hensify.^348^ These agents are either actively or passively targeted to enhance anticancer efficiency to reduce side effects. The reduced toxicity results from their ability to preferentially accumulate at the tumor site and limit off-target side effects.

**Table 2:** Representative smart nanoparticles that have been clinically approved for cancer diagnosis and treatment

Name	Particle type/drug	Approved application/indication	Approval (year)	Investigated application/indication
Doxil Caelyx (Janssen)	Liposomal doxorubicin (PEGylated)	Ovarian cancer (secondary to platinum based therapies) HIV-associated Kaposi’s sarcoma (secondary to chemotherapy) Multiple myeloma (secondary)	FDA (1995) EMA (1996)	Various cancers including: solid malignancies, ovarian, breast, leukemia, lymphomas, prostate, metastatic, or liver
DaunoXome (Galen)	Liposomal daunorubicin (non-PEGylated)	HIV-associated Kaposi’s sarcoma (primary)	FDA (1996)	Various leukemias
Myocet (Teva UK)	Liposomal doxorubicin (non-PEGylated)	Treatment of metastatic breast cancer (primary)	EMA (2000)	Various cancers including: breast, lymphoma, or ovarian
Abraxane (Celgene)	Albumin-particle bound Paclitaxel	Advanced non-small cell lung cancer (surgery or radiation is not an option) Metastatic breast cancer (secondary) Metastatic pancreatic cancer (primary)	FDA (2005) EMA (2008)	Various cancers including: solid malignancies, breast, lymphomas, bladder, lung, pancreatic, head and neck, prostate, melanoma, or liver
Marqibo (Spectrum)	Liposomal vincristine (non-PEGylated)	Philadelphia chromosome-negative acute lymphoblastic leukemia (tertiary)	FDA (2012)	Various cancers including: lymphoma, brain, leukemia, or melanoma
MEPACT (Millennium)	Liposomal mifamurtide (non-PEGylated)	Treatment for osteosarcoma (primary following surgery)	EMA (2009)	Osteosarcomas
Onivyde MM-398 (Merrimack)	Liposomal irinotecan (PEGylated)	Metastatic pancreatic cancer (secondary)	FDA (2015)	Various cancers including: solid malignancies, breast, pancreatic, sarcomas, or brain
Vyxeos CPX-351 (Jazz Pharmaceuticals)	Liposomal formulation of cytarabine: daunorubicin (5:1 M ratio)	Acute myeloid leukemia	FDA (2017) EMA (2018)	Various leukemias
Nbtxr3 Hensify (Nanobiotix)	Hafnium oxide nanoparticles stimulated with external radiation to enhance tumor cell death via electron production	Locally advanced squamous cell carcinoma	CE (Conformité Européene) Mark (2019)	Locally advanced soft tissue sarcoma
Feraheme (AMAG) Rienso (Takeda) Ferumoxytol	Iron polyglucose sorbitol carboxymethyl ether colloid	Iron deficiency in patients with chronic kidney disease	FDA (2009)	Iron deficient anemia Imaging: brain metastases, lymph node metastases, neuroinflammation in epilepsy, head and neck cancer, myocardial infarction, or multiple sclerosis
Definity (Lantheus Medical Imaging)	Perflutren lipid microspheres	Ultrasound contrast agent	FDA (2001)	Ultrasound enhancement for: liver or breast or intraocular or pancreatic tumors, pulmonary diseases, heart function, transcranial injuries, strokes, or liver cirrhosis
NanoTherm (Magforce Nanotech AG)	Amino silane-coated iron oxide NPs in magnetite form	Local ablation in glioblastoma	EMA (2010)	Thermal ablation, hyperthermia therapy. Local ablation in glioblastoma
Sienna (Endomagnetics Ltd)	Carboxydextran coated iron oxide NPs	Detection of cancerous sentinel lymphnodes in breast cancer; rectal cancer	EMA (2011)	Detection of cancerous sentinel lymphnodes in breast cancer; Rectal cancer
Venofer (AmericanRegent)	Iron sucrose colloid	Epithelial ovarian cancer; Gynecologic cancer; the treatment of iron deficiency anemia in adult patients with chronic kidney disease	FDA (2010)	Anemia; Pregnancy; Postural orthostatic tachycardia syndrome; Chronic heart failure; Inflammatory bowel disease; Postpartum anemia; Surgical intervention; Hip fracture; Hematological malignancies; Renal failure; Restless legs syndrome; Perioperative blood conservation; Colorectal neoplasm; Puerperal disorders; Critical illness; Hypertension; Premature birth
Injectafter Ferinject (Vifor)	Iron carboxymaltose colloid	Iron deficiency anemia in pancreatic cancer; cancer and chemotherapy related anemia; metastatic colorectal cancer; solid cancer metastatic disease	FDA (2013)	Fibromyalgia; Heart failure; Restless legs syndrome; Postoperative anemia; CKD; Colorectal neoplasm; Inflammatory bowel disease; Thrombocytosis; Postpartum anemia; Diabetes mellitus

Due to the success of these smart nanoparticles in the clinic and commercial field, enormous efforts continue to explore nanomedicines and developing new smart nanoparticles for clinically disquisitive trials.^287^ Therefore, the current clinical trials of smart nanoparticles for cancer diagnosis and treatment are also reviewed (Table 3). The list shows a variety of representative nanoparticle systems that have already been active or working in clinical trials. Some of these systems are liposomes, many of which have similar design characteristics to approved liposome systems (e.g., nontargeted, PEGylated, non-PEGylated, or encapsulate of a single drug). For example, VYEXOS/CPX‐351 is a combination therapy that encapsulates a synergistic ratio of two anticancer drugs (cytarabine and daunorubicin).^349^ The aim is to use the control of circulation and biological distribution of smart nanoparticles to target the delivery of highly toxic anticancer drugs. There are also many other smart nanoparticle delivery systems in clinical trials for cancer therapy, such as nanoparticle systems that target and stimulate responses. Despite the massive potential of nanoparticles for future cancer diagnostic and treatment, they are still at the relatively preliminary stages of clinical applications. A large number of challenges remain to be addressed to accelerate their further translations.

**Table 3:** Smart nanoparticles in clinical trials for cancer diagnosis and treatment

Name	Company	Particle type/drug	Investigated application/indication	Phase	ClinicalTrials.gov identifiers	Study start	Study completion	Status
Promitil	Lipomedix Pharmaceuticals	PEGylated liposomal mitomycin-C	Solid tumors	I	NCT01705002	2012.10	2018.6	Completed
				I	NCT03823989	2019.1	2021.6	Recruiting
Oncoprex	Genprex	FUS1 (TUSC2) encapsulated liposome	Lung cancer	I/II	NCT01455389	2014.2	2021.6	Active, not recruiting
Halaven E7389-LF	Eisai	Liposomal eribulin mesylate	Solid tumors	I	NCT01945710	2012.12	2016.5	Completed
				I	NCT03207672	2017.8	2021.4	Recruiting
Mitoxantrone hydrochloride liposome	CSPC ZhongQi Pharmaceutical Technology	Mitoxantrone liposome	Lymphoma and breast cancer	II	NCT02596373	2015.6	2018.9	Recruiting
				II	NCT02597387	2015.8	2018.10	Recruiting
				I	NCT02595242	2015.6	2017.6	Withdrawn
siRNA-EphA2-DOPC		siRNA liposome for EphA2 knockdown	Solid tumors	I	NCT01591356	2015.7	2020.7	Recruiting
PNT2258	ProNAi Therapeutics	Proprietary single-stranded DNAi (PNT100) encapsulated in lipid nanoparticles	Lymphomas	II	NCT02378038	2015.8	2016.6	Terminated
				II	NCT01733238	2012.11	2016.8	Completed
DCR-MYC	Dicerna Pharmaceuticals	DsiRNA lipid nanoparticle for NYC oncogene silencing	Solid tumors, multiple myeloma, lymphoma, or hepatocellular carcinoma	I	NCT02110563	2014.4	2016.11	Terminated
				I/II	NCT02314052	2015.1	2016.10	Terminated
SGT-53	SynerGene Therapeutics	Cationic liposome with Anti-transferrin receptor antibody, encapsulating wildtype p53 sequence	Glioblastoma, solid tumors, or pancreatic cancer	I	NCT02354547	2014.12	2021.11	Recruiting
				II	NCT02340156	2014.4	2018.11	Recruiting
				I	NCT00470613	2008.2	2016.12	Completed
				I	NCT03554707	2021.6	2023.12	Not yet recruiting
SGT-94	SynerGene Therapeutics	RB94 plasmid DNA in a liposome with anti-transferrin receptor antibody	Solid tumors	I	NCT01517464	2012.1	2015.12	Completed
MRX34	Mirna Therapeutics	Double-stranded RNA mimic of miR-34 encapsulated in liposomes	Liver cancer	I	NCT01829971	2013.4	2017.5	Terminated
				I	NCT02862145	2016.8	2017.12	Withdrawn
AZD2811	AstraZeneca with BIND Therapeutics	Aurora B kinase inhibitor in BIND therapeutics polymer particle accurin platform	Advanced solid tumors	I	NCT02579226	2015.10	2020.4	Completed
				II	NCT03366675	2017.12	2018.10	Terminated
				I	NCT03217838	2017.7	2022.3	recruiting
BIND-014	BIND Therapeutics	PSMA targeted (via ACUPA) docetaxel, PEG-PLGA or PLA–PEG particle	Prostate, metastatic, non-small cell lung, cervical, head and neck, or KRAS positive lung cancers	II	NCT02479178	2015.6	2020.1	Terminated
				II	NCT02283320	2014.9	2016.4	Completed
				II	NCT01812746	2013.4	2016.4	Completed
				II	NCT01792479	2013.4	2016.4	Completed
				I	NCT01300533	2011.1	2016.2	Completed
NC-6004 Nanoplatin	Nanocarrier	Polyamino acid, PEG, and cisplatin derivative micellar nanoparticle	Advanced solid tumors, lung, biliary, bladder, or pancreatic cancers	I/II	NCT02240238	2014.5	2019.5	Completed
NC-4016 DACH-Platin micelle	Nanocarrier	Polyamino acid, PEG, and oxaliplatin micellar nanoparticle	Advanced solid tumors or lymphomas	I	NCT03168035	2013.11	2017.4	Completed
NK105	Nippon Kayaku	Paclitaxel micelle	Breast cancer	III	NCT01644890	2012.7	2017.1	Completed
CriPec	Cristal Therapeutics	Docetaxel micelles	Solid tumors, ovarian cancer	I	NCT02442531	2015.8	2018.7	Completed
				I	NCT03712423	2018.4	2020.5	Completed
				II	NCT03742713	2018.10	2020.12	Completed
CRLX301	Cerulean	Cyclodextrin based nanoparticle-docetaxel conjugate	Dose escalation study in advanced solid tumors	I/II	NCT02380677	2015.44	2017.10	Terminated
ABI-009	Aadi with Celgene	Albumin bound rapamycin	Bladder cancer, PEComa, or pulmonary arterial hypertension	I/II	NCT02009332	2014.4	2019.12	Completed
				II	NCT03670030	2018.11	2021.11	Recruiting
				I	NCT03646240	2018.7	2021.12	Recruiting
				I	NCT03190174	2017.8	2021.4	Recruiting
				I	NCT00635284	2007.12	2011.6	Completed
				II	NCT03439462	2018.7	2021.12	Recruiting
				II	NCT03463265	2018.8	2021.7	Recruiting
ABI-011	NantBioScience	Albumin bound thiocolchicine analog (IDN 5405)	Solid tumors or lymphomas	I	NCT02582827	2017.11	2019.8	Withdrawn
Cornell Dots		Silica nanoparticles with a NIR fluorophore, PEG coating, and a^124^I radiolabeled cRGDY targeting peptide	Imaging of melanoma and malignant brain tumors	I	NCT03465618	2018.3	2022.3	Recruiting
				II	NCT02106598	2014.4	2021.4	Recruiting
Magnablate		Iron nanoparticles	Thermal ablation for prostate cancer	0	NCT02033447	2013.12	2015.1	Completed

## Conclusion and future perspective

Smart nanoparticles can carry and release anticancer medications at the specific areas to precisely treat cancers. The destiny of the drug-carrying nanocarriers remains a worry, though. Conventional nanoparticles can collect in the lungs, spleen, kidneys, liver, and heart, depending on their chemical composition, size, shape, specific surface area, surface charge, and presence or absence of a shell around them. The bulk of nanoparticles collect in the body’s important organs rather than being eliminated from the body. Toxicology is the result of this deposition and is a major obstacle to the success of smart nanoparticles. Animals have been used in a large number of in vitro and in vivo toxicity studies, but there have only been a small number of human investigations. It is yet unknown how far toxicity study research will go.

The main obstacle to the commercialization of smart nanoparticles is receiving FDA or other regulatory agencies’ approval. Although there are many products in the works, there are very few smart nanoparticles-based anti-cancer medications that have received FDA approval 27 years after the first one, Doxil, was initially disclosed in 1995. Manufacturers must demonstrate the goods’ short- and long-term safety and efficacy for the human body in order to receive regulatory clearance. Therefore, launching a product with all the required procedures is very time-consuming and labor-intensive. Sometimes the clearance process is made more difficult by the lack of specific rules. To remove these obstacles, contributions from the academic community, business community, and regulatory agencies are required.

Thankfully, the advancement of artificial intelligence paves the way for the construction of intelligent nanoparticles by making certain circumstances more conducive. Several different kinds of intelligent nanoparticles may be manufactured with the use of artificial intelligence. In today’s world of current pharmacological and therapeutic nanoparticle discovery, computer-aided nanoparticle design encompasses a wide variety of theoretical and computational methodologies. Methods of computer-aided nanoparticle design have been significant in the production of medications that are either now used in clinical practice or are undergoing clinical studies. Because of this, computer-aided nanoparticle design is a mix of numerous theoretical and computational fields, such as molecular modeling, cheminformatics, theoretical chemistry, and a number of others. Molecular docking, dynamics, quantitative structure-activity relationships (QSAR), and similarity searching are just a few of the many computational methods that are utilized in computer-aided nanoparticle design. These methodologies have been utilized for a number of years. Despite this, these strategies are continuously being developed and perfected. In addition, the development of computer-aided nanoparticle design is being propelled forward by a number of cutting-edge ideas and methods now being explored. Examples of the second category include “big data,” artificial intelligence, machine learning, and deep learning, amongst others. In the process of drug discovery, every new method as well as traditional ones are being employed in conjunction with emerging trends like polypharmacology and drug repurposing. In the same way as other multidisciplinary methods, computer-aided nanoparticle design faces a number of challenges. These challenges include not only the refinement of the theoretical basis, but also the rational application of the technology (while being aware of its limitations), as well as the education and training of those who will be utilizing the technology.

In conclusion, a variety of smart nanoparticles are being used or have the potential to be exploited as drug delivery systems for advanced cancer therapies. Due to their special qualities, clinicians are now able to provide new treatments or add them as supplements to already-effective therapies. As this review has demonstrated, smart nanoparticles have shown promise for developing safer and more effective cancer therapies, including targeted drug delivery, stimuli-responsive drug release, and co-delivery of combinational drugs. These capabilities may support long-term perspectives and the creation of novel cancer treatment approaches. Several novel materials are now being developed and have shown significant promise, which is generating optimism for the new therapeutic choices, even though some of these smart nanoparticles haven’t been successful throughout the clinical translation.
